# The *Polycomb* group protein MEDEA controls cell proliferation and embryonic patterning in *Arabidopsis*

**DOI:** 10.1016/j.devcel.2021.06.004

**Published:** 2021-07-12

**Authors:** Sara Simonini, Marian Bemer, Stefano Bencivenga, Valeria Gagliardini, Nuno D. Pires, Bénédicte Desvoyes, Eric van der Graaff, Crisanto Gutierrez, Ueli Grossniklaus

**Affiliations:** 1 Department of Plant and Microbial Biology & Zurich-Basel Plant Science Center, University of Zurich, Zollikerstrasse 107, 8008 Zurich, Switzerland; 2 Centro de Biología Molecular Severo Ochoa CSIC-UAM, Nicolás Cabrera 1, Cantoblanco 28049, Madrid, Spain; 3 BIOSS Centre for Biological Signaling Studies, Faculty of Biology, Albert-Ludwigs-Universität Freiburg, Schänzlestrasse 1, 79104 Freiburg, Germany

**Keywords:** *Arabidopsis*, cell proliferation, cyclin, embryonic patterning, evolutionary conservation, H3K27me3, *MEDEA*, plant development, *Polycomb* group, PRC2

## Abstract

Establishing the embryonic body plan of multicellular organisms relies on precisely orchestrated cell divisions coupled with pattern formation, which, in animals, are regulated by *Polycomb* group (PcG) proteins. The conserved *Polycomb* Repressive Complex 2 (PRC2) mediates H3K27 trimethylation and comes in different flavors in *Arabidopsis*. The PRC2 catalytic subunit MEDEA is required for seed development; however, a role for PRC2 in embryonic patterning has been dismissed. Here, we demonstrate that embryos derived from *medea* eggs abort because *MEDEA* is required for patterning and cell lineage determination in the early embryo. Similar to PcG proteins in mammals, *MEDEA* regulates embryonic patterning and growth by controlling cell-cycle progression through repression of *CYCD1;1,* which encodes a core cell-cycle component. Thus, *Arabidopsis* embryogenesis is epigenetically regulated by PcG proteins, revealing that the PRC2-dependent modulation of cell-cycle progression was independently recruited to control embryonic cell proliferation and patterning in animals and plants.

## Highlights

• *MEDEA*, a *Polycomb* Repressive Complex 2 (PRC2) subunit, is required in embryo and endosperm
• *MEDEA* links cell proliferation and differentiation during embryonic pattern formation
• The PRC2 protein *MEDEA* directly regulates core cell-cycle components, i.e., cyclin *CYCD1;1*
• Body plan and cell proliferation are epigenetically regulated in both animals and plants

## Graphical abstract

**Figure:**
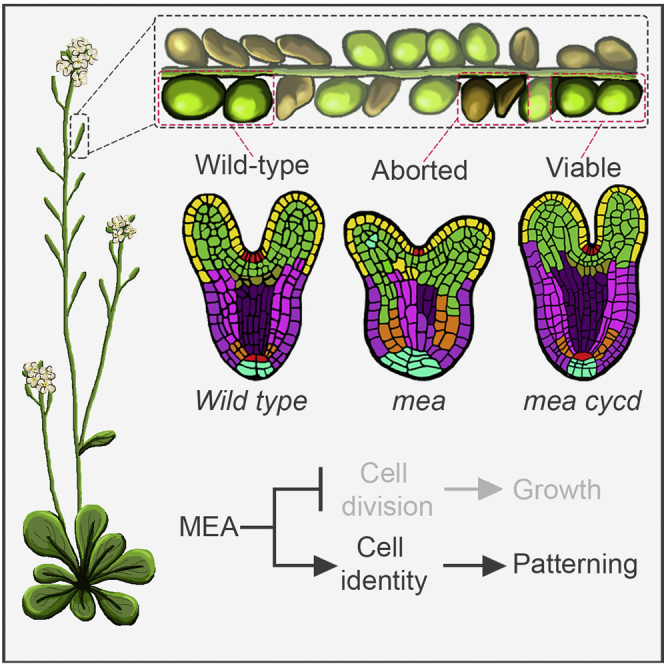

## Teaser

In animals, *Polycomb* group (PcG) proteins are essential for embryogenesis. Simonini et al. show that growth and patterning of plant embryos are also epigenetically regulated by PcG proteins, with the PRC2 histone methyltransferase *MEDEA* directly repressing the core cell-cycle component *CYCD1;1* in the *Arabidopsis* embryo.

## Introduction

A fundamental question in developmental biology is how cells acquire and maintain their identity over time. Most cell types are specified during embryogenesis (Stent, 1985; McDole et al., 2018) and result from synergistic interactions between growth and differentiation. Epigenetic regulation of gene expression is key to control cell proliferation preventing premature cell-identity acquisition. The *Polycomb* group (PcG) proteins play an important role in maintaining cell identity by silencing target genes, including pluripotency factors, whereas their deregulation is associated with cancer (Laugesen et al., 2016; Loubiere et al., 2019). To achieve this coordination, PcG proteins of *Drosophila* and vertebrates directly repress the expression of a wide variety of cell-cycle genes, including several core cell-cycle components, such as D- and A-type cyclins (Martinez et al., 2006; Iovino et al., 2013; Su et al., 2015; von Schimmelmann et al., 2016; Adhikari and Davie, 2020), which mediate entry and progression through S-phase, respectively (Sherr, 1995; Geng et al., 2001; Bulankova et al., 2013).

*Polycomb* Repressive Complex 2 (PRC2), a multi-subunit complex that is highly conserved from animals to plants, is a key player in the control of cell proliferation, cell fate determination, and cell differentiation at various developmental stages in multicellular organisms (Grossniklaus and Paro, 2014). The trimethylation of histone H3 at lysine 27 (H3K27me3) is the hallmark of PRC2 activity and is typically found at transcriptionally silenced loci. While mutations affecting PRC2 subunits lead to embryo lethality in animals (Faust et al., 1995; O’Carroll et al., 2001; Oktaba et al., 2008; Pasini et al., 2004; Grosswendt et al., 2020), plants lacking PRC2 components do not show severe embryonic phenotypes and most produce viable offspring (Bouyer et al., 2011; Chanvivattana et al., 2004; Kinoshita et al., 2001). This is also because plants possess several different PRC2 complexes: EMF-PRC2 controls aspects of vegetative development; VRN-PRC2 regulates flowering time and the plants’ response to vernalization; and FIS-PRC2 has a specific role in reproduction, particularly in female gametophyte, endosperm, and seed development (Grossniklaus and Paro, 2014; Hugues et al., 2020). Single and double mutants for plant PRC2 subunits are viable, with the exception of mutations affecting components of FIS-PRC2, i.e., *MEA* (Grossniklaus et al., 1998), *FERTILIZATION-INDEPENDENT ENDOSPERM* (*FIE*) (Ohad et al., 1999), *FERTILIZATION-INDEPENDENT SEED2* (*FIS2*) (Luo et al., 1999), and *MULTICOPY SUPPRESSOR of IRA1* (*MSI1*) (Köhler et al., 2003a). Seeds inheriting maternal mutant alleles of these *FIS*-class genes abort due to a failure in endosperm cellularization and embryonic arrest, regardless of the paternal genotype. This maternal effect is observed because *MEA* and *FIS2* are regulated by genomic imprinting, leading to parent-of-origin-dependent allelic expression (Jullien et al., 2006; Kinoshita et al., 1999; Vielle-Calzada et al., 1999). This form of epigenetic gene regulation evolved independently in seed plants and mammals (Pires and Grossniklaus, 2014); in the latter, it plays a prominent role in the placenta and is required for normal embryonic development (Barlow and Bartolomei, 2014; Ferguson-Smith, 2011).

Although mutants affecting FIS-PRC2 components produce embryos with increased cell proliferation and disorganized division planes (Grossniklaus et al., 1998; Ohad et al., 1999), embryo abortion was considered to indirectly result from defects in the endosperm (Kinoshita et al., 1999; Scott et al., 1998). In support of this conclusion is the observation that embryos lacking the two PRC2 methyltransferases CURLY LEAF (CLF) and SWINGER (SWN) develop normally (Chanvivattana et al., 2004), that *fie* homozygous seeds do not exhibit severe morphological defects (Bouyer et al., 2011), and embryo rescue of homozygous *mea* seeds produces wild type (WT)-looking, albeit sterile, plants (Grossniklaus et al., 1998). All these observations have led to the conclusion that FIS-PRC2 is not essential for embryonic development (Kiyosue et al., 1999; Leroy et al., 2007) and that, unlike in animals, PRC2 does not play a major role in regulating plant embryogenesis. However, although there is some overlap in expression with *CLF* or *SWN* (Spillane et al., 2007), *MEA* is the major methyltransferase expressed in the early embryo. Moreover, in none of the previous studies could the genotype of embryo and endosperm be uncoupled nor young embryos be isolated from the surrounding endosperm and maternal seed coat. This is because of the complexity of plant reproduction involving two fertilization events. In plants, gametes are produced by the multicellular male and female gametophytes, the pollen and embryo sac, respectively. These haploid structures are formed inside the reproductive organs through mitotic divisions of the spores resulting from meiosis. In both pollen and embryo sac, two gametes are formed and, because the gametophytes are typically derived from a single spore, the gamete pairs are genetically identical. When the pollen delivers the two sperm cells to the embryo sac, double fertilization occurs, whereby the female gametes, egg and central cell, each fuse with one sperm to develop into embryo and endosperm, respectively. After fertilization, the developing embryo is surrounded by proliferating endosperm and the maternal seed coat. For many analyses, it has proven difficult to isolate a sufficient number of embryos from the seed while preserving their cellular integrity. Thus, whole seeds were typically employed, thereby losing the spatial and cellular resolution required to investigate certain aspects of embryogenesis independently of the influence of the endosperm.

Here, we adopt a genetic strategy to uncouple the fertilization events of egg and central cell, in order to generate seeds where embryo and endosperm have discordant genotypes, thereby allowing us to explore the spatial requirement for *MEA* function during seed development. We demonstrate that *mea-*deficient endosperm is capable of sustaining embryonic growth and that embryos derived from *mea* egg cells (referred to as *mea* embryos) abort regardless of the genotype of the endosperm because *MEA* is required for the establishment of embryonic patterning and cell-lineage differentiation. Thus, also in *Arabidopsis*, embryonic patterning is epigenetically controlled by maternally expressed PcG proteins. By using isolated embryos at the early developmental stages, we could characterize molecular signatures that were undetectable in whole seeds. We show that *MEA* controls embryonic growth by repressing the transcription of the core cell-cycle component *CYCD1;1*, which encodes a D-type cyclin. The identification of *CYCD1;1* as a target of MEA provides the first conceptual link between an underlying molecular mechanism and the mutant phenotypes of *fis*-class mutants, i.e., defects in cell proliferation that were described over two decades ago. Thus, our work provides mechanistic insights linking cell-cycle regulation and patterning in *Arabidopsis*, thereby revealing a cross-kingdom conservation of PRC2 function in animals and plants.

## Results

### *MEA* is required for embryogenesis in *Arabidopsis*

**Figure 1:**
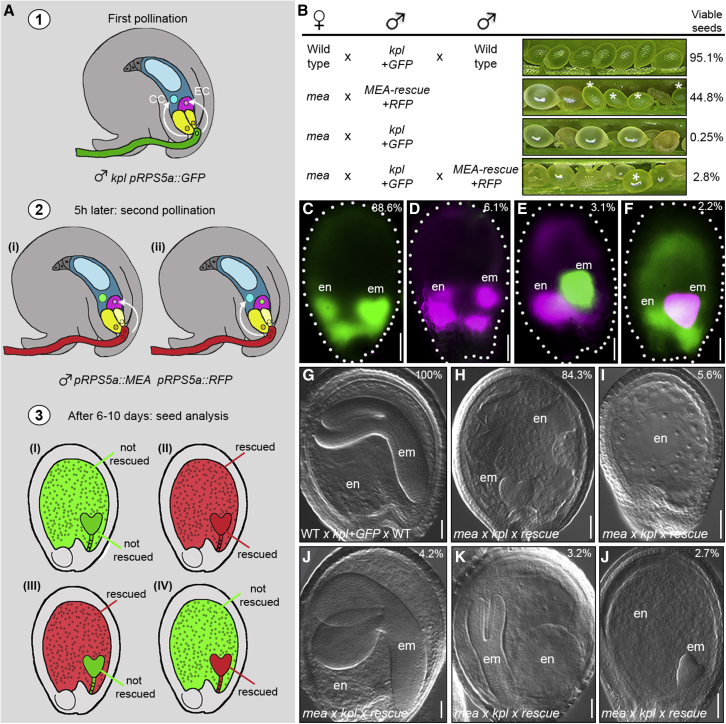
Genetically uncoupling embryo and endosperm development reveals the requirement of *MEA* for embryogenesis (A) Schematic representation of the strategy adopted to generate seeds with embryo and endosperm having discordant genotypes. EC, egg cell; CC, central cell. (B) Set of crosses performed, with opened siliques showing developing seeds. Asterisks indicate developing seeds among aborting ones. (C–F) Fluorescent microscopy images of seeds derived from the double pollination experiment: no rescue (C), complete rescue (D), endosperm only rescue (E), and embryo only rescue (F). (G–L) Clearing of seeds of the WT *× kpl+GFP* × WT cross (G) and the five phenotypic classes (H–L) observed in *mea × kpl+GFP × MEA−rescue+RFP* crosses: *mea*-like (H), endosperm only (I), *MEA*-rescued (J), WT-looking embryo and *mea* endosperm (K), and abnormal embryo and WT-looking endosperm (J). Top right corner: percentage of seeds showing the phenotype. em, embryo; en, endosperm. Scale bar, 50 μm

In addition to expression in the endosperm, *MEA* transcript was detected in embryos up to the torpedo stage (Spillane et al., 2007; Vielle-Calzada et al., 1999). Embryos that develop from fertilized *mea* eggs are larger than WT and arrest around the heart stage (Grossniklaus et al., 1998). So far, it was not possible to separate direct effects of loss of *MEA* in the embryo from indirect ones resulting from aberrant endosperm development. To distinguish direct and indirect effects, we aimed to generate seeds with genetically distinct embryo and endosperm, in which only one of the two develops in absence of *MEA* activity, i.e., seeds where a WT embryo grows surrounded by *mea*-defective endosperm and, vice versa, seeds where *mea* embryos develop surrounded by WT endosperm. To obtain such seeds, we pollinated pistils consecutively with two genetically distinct pollen donors. The first fertilization event was achieved with pollen that contains only a single sperm cell and, thus, fertilizes either the egg or the central cell (Figure 1A, step 1). Incomplete double fertilization allows the embryo sac to attract a second pollen tube (Maruyama et al., 2013) (Figure 1A, step 2). The second fertilization event involves genetically distinct pollen, and thus the genotype of endosperm and embryo will differ in a fraction of the seeds (Figure 1A, step 3). To distinguish and isolate the seeds that develop with discordant embryo and endosperm genotypes, we introduced the fluorescent proteins GFP or RFP into the different pollen donors (Figure 1A). Both fluorescent reporters are expressed under the *pRPS5a* promoter driving expression in actively proliferating tissues, including embryo and endosperm (Weijers et al., 2001).

In our experiment, *pRPS5a::GFP* marked the first fertilization event and *pRPS5a::RFP* the second one. To perform the first single fertilization event, we used as pollen donor the *kokopelli* (*kpl*) mutant, which produces some pollen with only a single sperm (Ron et al., 2010) and carries the *pRPS5a::GFP* marker (referred as *kpl+GFP*). In the pollination events of WT pistils made only with *kpl+GFP* pollen, 80.8% of the seeds underwent double fertilization, producing viable seeds (n = 631; Figure S1A). The remaining seeds aborted and had either some endosperm nuclei but no embryonic structures or contained arrested embryos around the globular stage without any endosperm. Thus, these aborted seeds were the result of a single fertilization event of either the egg cell (embryo without endosperm) or the central cell (endosperm without embryo). We then repeated the pollination of WT pistils with the *kpl+GFP* pollen, followed by a second pollination with pollen of *pRPS5a::RFP* plants. Plants carrying the *pRPS5a::RFP* construct produce WT pollen with two functional sperm cells. The percentage of viable seeds after two consecutive pollination events of WT pistils increased to 95.1% (n = 645, Figures 1B and S1A) from 80.8%, when only the *kpl+GFP* pollen was used. Thus, the second pollination with *pRPS5a::RFP* pollen allowed seed formation from most of those embryo sacs that had experienced an initial single fertilization, thereby rescuing seed abortion. We then characterized the composition of the seeds originating from two consecutive pollinations, using the GFP and RFP markers. Four classes of seeds could be observed (n = 837; Figures 1C–1F): (1) GFP-positive endosperm and embryo (88.6%, Figure 1C), derived from a unique pollination event with a two-sperm-celled *kpl+GFP* pollen; (2) RFP-positive endosperm and embryo (6.1%, Figure 1D), derived from double fertilization by *pRPS5a::RFP* pollen of those few ovules that were not fertilized in the first round by *kpl+GFP* pollen; (3) RFP-positive endosperm and GFP-positive embryo (3.1%, Figure 1E); and (4) GFP-positive endosperm and RFP-positive embryo (2.2%, Figure 1F). In these last two classes, the distinct fluorescent profiles of embryo and the endosperm indicate discordance of their genotype.

We then applied this strategy to generate seeds, in which only the embryo—or the endosperm—develops in the absence of *MEA* activity. To this aim, we used either *mea* homozygous individuals that, albeit at very low frequency, spontaneously developed from *mea/MEA* heterozygous plants or *mea/mea MEA-GR* plants that were not treated with dexamethasone (DEX) such that the MEA-GR fusion protein remained inactive (Pires et al., 2016). To provide *MEA* activity, we generated a rescue construct, *pRPS5a::MEA*, allowing paternal expression of *MEA* soon after fertilization. *MEA* is an imprinted gene, with only the maternal *MEA* being expressed in the fertilization products, while the paternal allele is silenced (Gehring et al., 2009, 2006; Grossniklaus et al., 1998; Jullien et al., 2006; Vielle-Calzada et al., 1999). In the line we used, expression of the paternally introduced *pRPS5a::MEA* construct could rescue 44.8% of the *mea* homozygous seeds (n = 665, Figures 1B and S1A), confirming that embryo and endosperm can develop normally if *MEA* activity is provided immediately following fertilization. We coupled the *pRPS5a::MEA* rescue construct with the *pRPS5a::RFP* marker (referred to as *MEA-rescue+RFP*) in order to identify seeds with discordant genotypes of embryo and endosperm. The genotypes were assessed by detection of RFP, either through microscopy or genotyping. *MEA*-rescued embryos that develop surrounded by *mea*-defective endosperm have RFP-positive embryos and GFP-positive endosperm, whereas *mea* embryos that grow in presence of *MEA*-rescued endosperm contain a GFP-positive embryo and RFP-positive endosperm.

We first pollinated *mea* homozygous pistils with only *kpl+GFP* pollen and, as expected, did not observe any rescue of seed abortion because the paternal *MEA* allele is inactive (n = 692; Figures 1B and S1A). The progeny of the *mea x kpl+GFP* cross was entirely made up of shrunken, dark brown seeds that did not germinate on culture media or soil. We then performed consecutive pollinations of *mea* homozygous plants with pollen from *kpl+GFP*, followed by *MEA-rescue+RFP* pollen. In the progeny of this double pollination, we detected few normally shaped and mature seeds (6.0%, n = 1,082), from which healthy but partially sterile plants developed (42 plants from 65 WT-looking seeds, 65%). All these individuals tested positive when genotyped for the presence of RFP and negative for GFP, confirming that they originated from *MEA*-rescued embryos. We repeated the two consecutive pollinations with the aim to isolate rescued seeds at an earlier stage so the genotype of the endosperm could also be analyzed. Around eight days after pollination, when WT control seeds showed green embryos at the bent-cotyledon stage, *mea* siliques showed three classes of seeds (n = 1,056; 721 from *mea/mea MEA::GR* not DEX induced, and 335 from spontaneous *mea/mea* homozygotes): (1) *mea* seeds (91.8%); (2) WT-like seeds (5.4%); and (3) *mea*-like seeds of enlarged, round, and translucent appearance but containing a WT-looking embryo at the walking-stick stage (2.8%, Figure 1B). Detection of the RFP-GFP signal was not conclusive for this class of seeds, as the *pRPS5a* is weakly expressed at this developmental stage and the embryo is rich in chlorophyll. Thus, we manually separated embryo and endosperm to genotype them individually by droplet digital PCR (ddPCR). Single-embryo ddPCR did not produce consistent results due to the low input of DNA; we thus pooled five embryos into one sample, confirming that all the walking stick embryos were RFP positive/GFP negative (n = 25, five samples with five embryos each) and, therefore, *MEA*-rescued. However, we failed to obtain consistent results for the genotype of the endosperm. Absence of *MEA* causes failure in proliferation and cellularization of the endosperm, which then remains in a liquid form (Kiyosue et al., 1999) and, thus, is challenging to collect. Therefore, we genotyped entire, single seeds by ddPCR, confirming that they carried both the GFP and RFP transgenes (n = 46; Figure S1D). Given that *kpl+GFP* alone does not rescue *mea* embryos and that the embryos tested positive for RFP, these seeds must have contained *mea* embryos carrying *MEA-rescue* construct (*MEA-rescue*+*RFP* sperm fertilized the egg cell) surrounded by *mea*-mutant endosperm (*kpl*+*GFP* sperm fertilized the central cell).

**Figure 2:**
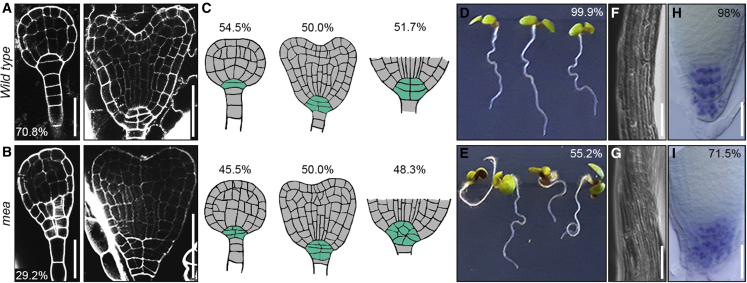
*mea* embryos develop severe morphological defects (A and B) mPS-PI staining of *mea/MEA* seeds showing WT (A) and aberrant (B) morphology at the globular (left image) and late heart stage (right image). (C) Schematic representation of embryos in *mea/MEA* plants with wild-type (top row) or *mea* (bottom row) phenotypes obtained using mPS-PI images as template. Late globular, heart, and the basal part of late heart stage embryos are shown from left to right. The columella/QC region is highlighted in turquoise. (D and E) WT (E) and *mea* homozygous (F) seedlings grown on vertical plates. (F and G) Magnification of epidermal cells of the primary root of WT (F) and *mea* homozygous (G) seedlings grown vertically. (H and I) Lugol staining of the primary root tip of WT (H) and *mea* homozygous (I) seedlings. Scale bars, 25 μm (A and B, left panels; H–J), 50 μm (A and B, right panels), 250 μm (F and G).

We morphologically characterized embryo and endosperm by clearing seeds derived from such double-pollination events of *mea* plants. 8 days after the consecutive pollinations, when WT seeds harbored embryos at the bent-cotyledon stage (Figure 1G), we observed five phenotypic classes (n = 1,235; Figures 1H–1L): (1) *mea*-looking seeds with embryos arrested around the heart stage and uncellularized endosperm (84.3%, Figure 1H); (2) seeds without visible embryos (5.6%, Figure 1I), originating from either single fertilization of the central cell or autonomous endosperm development; (3) WT-looking seeds (4.2%, Figure 1J) derived from double fertilization with *MEA-rescue+RFP* pollen; (4) seeds with WT-looking embryos at the walking-stick stage, surrounded by uncellularized, defective endosperm (3.2%, Figures 1L and S1C) derived from fertilization of the egg cell by a *MEA-rescue+RFP* sperm; and (5) seeds with abnormal embryos arrested around the heart stage and cellularized, WT-looking endosperm (2.7%, Figure 1K), originating from fertilization of the central cell by a *MEA-rescue+RFP* sperm. This last class of seeds originated from two single consecutive fertilization events, resulting in seeds harboring genetically distinct embryo and endosperm with only the endosperm carrying the *MEA-rescue* construct.

Taken together, these results demonstrate that embryo and endosperm development can be uncoupled in *mea*-mutant seeds and that the development of *mea* embryos arrests regardless of the genotype of the endosperm (Figure 1K). Our analysis revealed that the failure in endosperm proliferation and cellularization in *mea* seeds does not cause abortion of the embryo because *MEA*-rescued embryos, even if surrounded by *mea-*deficient endosperm, complete development and produce viable progeny (Figure S1C). Thus, *MEA* activity is autonomously required in the embryo for normal embryogenesis to take place.

### *mea* embryos display patterning defects, particularly in the root apical meristem

Embryos originating from *mea* egg cells develop as disorganized mass of highly vacuolated cells with small, asymmetric cotyledons and an enlarged root meristem (Grossniklaus et al., 1998). To characterize the defects at cellular resolution, we performed a modified pseudo-Schiff Propidium Iodide staining (mPS-PI) of siliques from *mea/MEA* heterozygotes (Figures 2A–2C and S2A–S2D), where a 1:1 segregation of WT and *mea* embryos is expected. At the globular stage, 29.2% of the embryos showed ectopic cell divisions in the basal part of the embryo, such that they became almond-shaped (n = 48; Figures 2A, 2B, S1A, and S1B). Disorganized and excessive cell divisions are characteristic for the basal part of *mea* embryos throughout development (Figures 2A–2C and S2A–S2D). At the late heart stage, when WT embryos contained five to seven cells in the columella and quiescent center (QC) region, this region contained between seven and 18 cells in *mea* embryos (n = 120; Figures 2C and S2D). Consequently, the pyramidal organization typical of this part of WT root meristems was replaced by a mass of globularly arranged cells in *mea* embryos (Figures 2C and S2B). Consistent with these root meristem defects, when grown on vertical plates, *mea* homozygous seedlings exhibited severe agravitropic growth and the primary root made loops, upward turns, and displayed twisted epidermal cells as well as an altered columella root tip organization (n = 76; Figures 2D–I, 2E).

**Figure 3:**
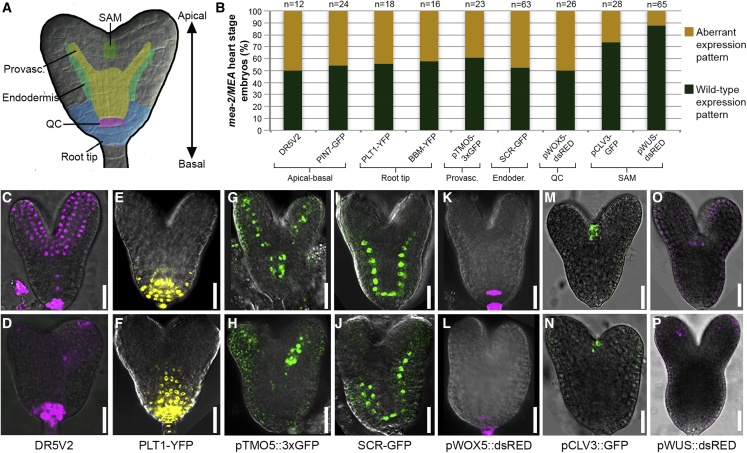
Embryonic patterning is affected in *mea* embryos (A) Schematic representation of the analyzed embryonic domains. (B) Percentage of embryos showing an altered expression pattern of the corresponding marker line in *mea/MEA* seeds. (C–P) Confocal images showing the expression patterns in WT embryos (upper row) and *mea*-like embryos (lower row) at late heart stage for DR5V2 (C and D), *pPLT1::PLT1-YFP* (E and F), *pTMO5::3xGFP* (G and H), *pSCR::SCR-GFP* (I and J), *pWOX5-dsRED* (K and L), *pCLV3::GFP* (M and N), and *pWUS::dsRED* (O and P). Scale bar, 20 μm

The morphological defects of *mea* embryos are reminiscent of mutants with compromised embryonic patterning (Jenik et al., 2007; Möller and Weijers, 2009). To verify this hypothesis, we crossed *mea/MEA* with a set of tissue-specific markers for distinct embryonic domains (Figure 3A): apical-basal patterning (DR5V2 and *pPIN7::PIN7-GFP*, Figures 3B–3D, S3A, and S3B), root tip architecture (*pPLT1::PLT1-YFP* and *pBBM::BBM-YFP*, Figures 3B, 3E, 3F, S3C, and S3D), provasculature (*pTMO5::3xGFP*, Figures 3B, 3G, 3H, and S3E), endodermis (*pSCR::SCR-GFP*, Figures 3B, 3I, 3J, and S3F), QC establishment (*pWOX5::dsRED*, Figures 3B, 3K, 3L, and S3G), and shoot apical meristem specification (SAM; *pWUS::dsRED* and *pCLV3::GFP*, Figures 3B, 3M, 3P, S3H, and S3I). We observed severely altered expression patterns of markers for different root regions, including expansion of the expression domain (*pPLT1::PLT1-YFP*, *pBBM*::*BBM-YFP*, *pPIN7-PIN7::GFP*, Figures 3E, 3F, and S3B–S3D), ectopic expression in a different embryonic region (DR5V2, *pTMO5::3xGFP*, Figures 3C, 3D, 3G, 3H, S3A, and S3E), and absence of the signal suggesting loss of cellular identity (*pSCR::SCR-GFP* and *pWOX5::dsRED*, Figures 3I, 3J, 3K, 3L, S3F, and S3G). For instance, in the embryonic root, where DR5V2 marks a restricted area of the root tip in WT embryos, the DR5V2 expression domain was expanded in *mea* embryos (Figures 3C, 3D, and S3A) while the QC marker *pWOX5::dsRED* was not expressed (Figures 3K, 3L, and S3G), in agreement with the observed defects in the root apical meristem. Severe polarity defects of *mea* embryos, predominantly along the apical-basal axis, were reflected by the aberrant expression pattern of various markers, including DR5V2, *pTMO5::3xGFP*, and *pPLT1::PLT1-YFP*. The ectopic expression of the *pPLT1::PLT1-YFP* in the upper half of the embryo (Figures 3E, 3F, and S3C) suggests an expansion of the root domain, whereas absence of the DR5V2 and *pTMO5::3xGFP* signals in the inner part of the embryo (Figures 3C, 3D, 3G, 3H, S3A, and S3E) indicates a dramatic underdevelopment of the provascular system. Apart from defects in apical-basal patterning (Figures 3C and 3D), radial patterning was also affected. This is best reflected by changes in the expression domains of *pPLT1::PLT1-YFP* (Figures 3E, 3F, and S3C) and *pTMO5::3xGFP* (Figures 3G, 3H, and S3E), which mark part of the provascular domain, and of *pSCR::SCR-GFP* that shows ectopic expression, particularly at early stages (Figure S3F), instead of a pattern restricted to the endodermis (Figures 3I, 3J, and S3F) and its precursors. Consistent with the fact that cotyledons in *mea* embryos are only mildly affected, the shoot apical meristem domain was properly specified with only a small percentage of embryos showing weak or ectopic marker expression (*pWUS::dsRED* and *pCLV3::GFP*, Figures 3M–3P, S3H, and S3I). For example, expression of the markers *pTMO5::3xGFP* (Figures 3G, 3H, and S3E) and *pSCR::SCR-GFP* (Figures 3I and S3F–S3J) was sometimes reduced in one of the cotyledons.

In summary, from early stages onwards, *mea* embryos display altered polarity, disturbed symmetry, and an abnormal specification of embryonic domains and tissues. These results strongly suggest that *MEA* is required for proper embryonic patterning at early stages of development. Thus, in *Arabidopsis*, the spatial and temporal definition of the embryonic body plan relies on PcG proteins as it does in animals (Margueron and Reinberg, 2011).

### Cell-cycle progression is compromised in *mea* embryos

**Figure 4:**
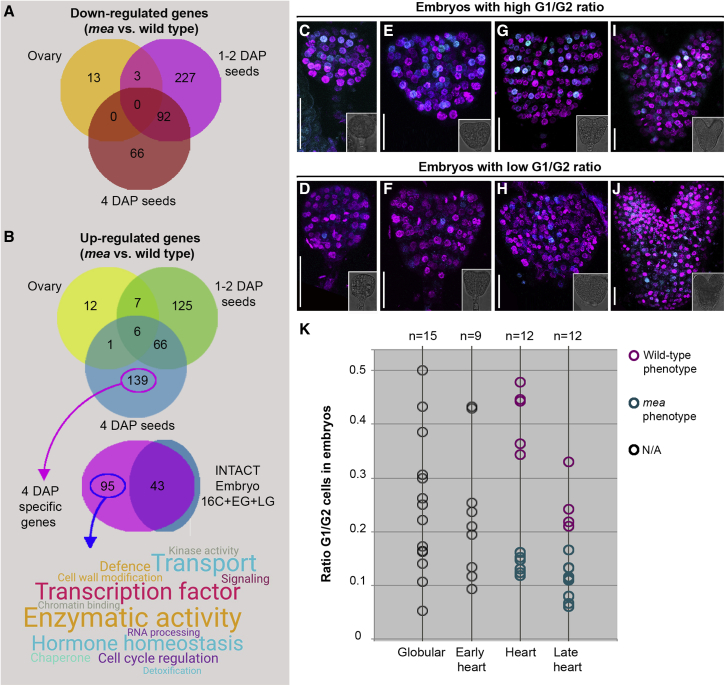
*mea* embryos display an accelerated cell cycle (A) Venn diagram depicting the sets of downregulated genes in transcriptomic analyses of *mea* homozygous versus WT ovaries/developing seeds. (B) Schematic representation of upregulated genes in transcriptomic datasets of *mea* homozygous versus WT ovaries/developing seeds, with Venn diagram (top) and word cloud of terms (bottom) for molecular function of 4DAP-specific genes. 16C, 16-cell embryos; EG, early globular embryos; LG, late globular embryos. (C–J) Confocal microscopy images of embryos of *mea/MEA* seeds expressing the PlaCCI triple cell-cycle marker line. Images show *CTD1-CFP* (G1) and *H3.1-RFP* (S+early G2) signals; the M-phase marker is not included. Inlets in bottom left corners: brightfield images of the embryos analyzed. (K) Quantification of the G1/G2 ratio in embryos of *mea/MEA* seeds. Pink circles are WT-looking embryos, turquoise circles are *mea*-like embryos; gray circles are embryos for which a phenotypic distinction was not possible. Scale bar, 20 μm

To identify the causative genes responsible for the patterning defects in *mea* embryos, we adopted a transcriptomics approach. Total RNA of *mea* homozygous and WT ovaries or seeds was collected at three time points: before fertilization (Ovary), one-two days after pollination (1-2DAP), and four days after pollination (4DAP). A total of 356 upregulated and 401 downregulated unique genes were represented in the three datasets combined (Figures 4A and 4B; Table S1). Among the upregulated genes, we found (1) factors known for their relationship with MEA, including the previously identified MEA targets *PHERES1* (*PHE1*) and *PHE2* (Kohler et al., 2003b), (2) *MEA* itself (Baroux et al., 2006), (3) a series of MADS-box transcription factor genes (*AGL28*, *AGL35*, *AGL36 AGL46*, *AGL64*, *AGL67*, and *PISTILLATA*), which are commonly found deregulated in *fis*-class mutant seeds (Zhang et al., 2018; Kradolfer et al., 2013), and (4) the nuclear factor *ADMETOS*, mutations in which were shown to partially suppress the *mea* phenotype (Kradolfer et al., 2013) (Table S1). Among the most highly represented proteins were (1) transcription factors (MADS-domain, basic HELIX-LOOP-HELIX, HOMEO-domain, and TEOSINTE BRANCHED1/CINCINNATA/PROLIFERATING CELL FACTOR [TCP] proteins); (2) factors involved in proteasome-mediated protein degradation (18 F-box proteins, five E3-ubiquitin ligases, and two SNW/SKI-INTERACTING PROTEINs [SKIPs]); and (3) components of hormonal pathways, predominantly of auxin, gibberellin, and jasmonic acid (Table S1). Interestingly, factors involved in DNA methylation, such as *DNA METHYLTRANSFERASE2* (*MET2*) and *MET3*, were also among the upregulated genes (Table S1). In agreement with the role of PRC2 proteins in epigenetic repression through H3K27me3 deposition (Cao et al., 2002), 43.5% of the upregulated genes were also enriched for H3K27me3 in the endosperm (Table S1; Moreno-Romero et al., 2016).

Taken together, these findings highlight the fundamental role of MEA in regulating the expression of factors that influence the DNA methylation landscape, transcriptional activity, hormone levels, and protein turnover.

Similarly, the vast majority of downregulated genes were involved in transcription, proteasome-mediated protein degradation, and hormonal homeostasis, particularly of auxin (Table S1). Eight out of the 23 *AUXIN RESPONSE FACTORs* (*ARFs*) encoded in the *Arabidopsis* genome with a known role in embryonic growth or endosperm development (*ARF12*, *ARF13*, *ARF14*, *ARF15*, *ARF20*, *ARF21*, *ARF22*, and *ARF23*; Hamann et al., 2002; Weijers et al., 2006; Rademacher et al., 2012) were downregulated in *mea* seeds. However, in contrast to the upregulated genes, DNA methylation was not represented and different biological processes, such as embryonic development and seed/endosperm development, were enriched (Table S1). Among the latter, we found *bHLH95/ZOUPI* (also known as *RETARDED EMBRYO GROWTH1*) and the subtilisin-like serine protease *ABNORMAL LEAF SHAPE1* (*ALE1/SBT1.4*), both of which are required for the formation of the embryonic cuticle (Tanaka et al., 2001; Kondou et al., 2008; Yang et al., 2008; Doll et al., 2020). The striking morphological similarities between *mea* embryos and embryos with *ALE1* misexpression (Doll et al., 2020) point to a possible defect in epidermis formation in *mea* embryos.

In summary, these data indicate a reduction in the abundance of embryo- and endosperm-expressed transcripts in *mea* seeds. Given that PRC2-mediated gene regulation typically mediates transcriptional repression (Cao et al., 2002), we speculate that the decrease of these transcripts is an indirect effect of aberrant embryo and endosperm development, rather than the direct transcriptional regulation by *MEA*.

To identify potential factors that are involved in the defects observed in *mea* embryos, we focused on genes showing significant upregulation in *mea* mutants compared with the WT (Table S1), because absence of PRC2 activity leads to transcriptional de-repression (Kirmizis et al., 2004; Köhler et al., 2003b). To enrich for potential targets responsible for the observed defects in *mea* embryos, we selected genes that were uniquely upregulated in the 4DAP dataset (Figure 4B), the time point when embryo-derived transcripts are technically detectable in samples using entire ovaries or seeds. A total of 139 upregulated genes were represented in the 4DAP dataset only (Figure 4B), with 95 of them showing no detectable expression in WT embryos at the 16-cell, early globular, and late globular stages (INTACT datasets (Palovaara et al., 2017); Figure 4B; Table S1). Manual annotation of the molecular functions of these genes (represented as word cloud, Figure 4B) revealed a predominant representation of factors involved in transcription, transport, enzymatic activity, hormone homeostasis, and regulation of the cell cycle (Figure 4B). We focused on the latter since *mea* embryos have more cells compared with the WT (Grossniklaus et al., 1998).

In order to measure the rate at which cells divide in *mea* embryos, we crossed *mea/MEA* plants with a triple cell-cycle marker line (PlaCCI, Desvoyes et al., 2020), enabling the simultaneous visualization of G1, S+early G2, and late G2+M phases. Two distinct classes of embryonic expression patterns were identified (Figures 4C–4K): (1) embryos in which many nuclei were in G1 (high G1/G2 ratio, i.e., CFP-positive/RFP-positive nuclei), and (2) embryos which had a low G1/G2 ratio. At heart and late heart stages, when *mea* embryos were clearly distinguishable from the WT, all embryos with a low G1/G2 ratio displayed the *mea* phenotype (Figures 4G–4K). As CFP is fused to the CTD1a protein, which is rapidly degraded upon entry into S phase, a low number of CFP-positive nuclei indicates that more cells have entered S phase and are, thus, committed to divide.

Thus, cell-cycle progression through G1 is accelerated in *mea* embryos, a function that has also been described for maternally expressed imprinted genes in mammals (Barlow and Bartolomei, 2014; Lau et al., 1994; Leighton et al., 1995; Wang et al., 2007). In summary, our results support a role for *MEA* in regulating the embryonic body plan through the control of cell division.

### Deregulation of a core cell-cycle component underlies the defects in *mea* embryos

Accelerated and disorganized, ectopic cell divisions arise from the deregulation of cell-cycle components (Gutierrez, 2009). D-type cyclins (CYCD) are conserved core constituents of the cell-cycle machinery that integrate cell division and tissue patterning by promoting the G1-S transition (Meijer and Murray, 2000). Altered CYCD levels are sufficient to induce cell division by shortening the G1 phase and to trigger formative division defects (Forzani et al., 2014; Sozzani et al., 2010), a phenotype we observed in *mea* embryos. Among the genes that fall under the class “regulation of cell cycle,” *CYCD1;1* was specifically upregulated at 4DAP in *mea* seeds (Table S1), and its increased expression in *mea* embryos was confirmed by ddPCR on RNA extracted from manually isolated embryos around the early globular stage (Figure S4A).

**Figure 5:**
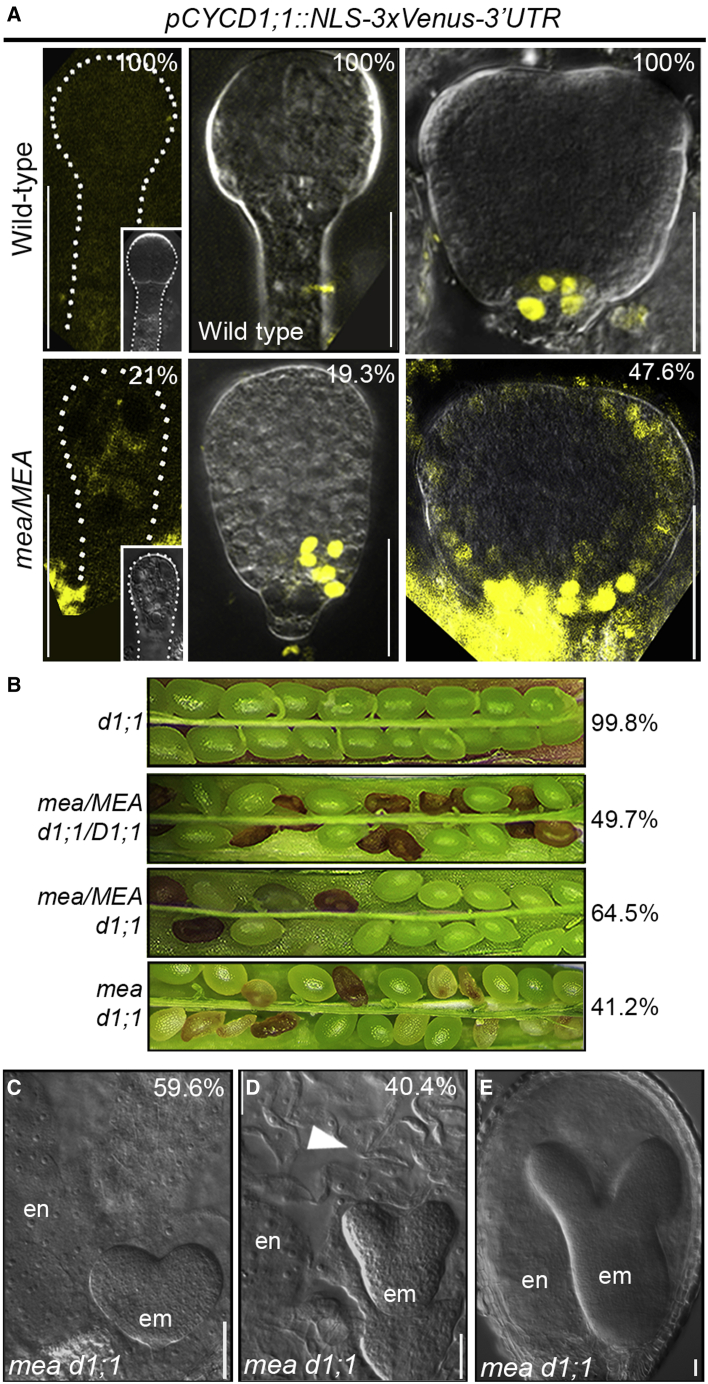
Ectopic expression of *CYCD1;1* is largely responsible for the morphological defects of *mea* embryos (A) Confocal microscopy images of embryos of *mea/MEA* plants showing expression of *pCYCD1;1::NLS-3xVenus-3’UTR* in WT (top) and *mea/MEA* (bottom) embryos at the 4–8-cell (left), globular (middle), and early heart (right) stage. Inlet in bottom right corners: brightfield images of the embryos analyzed. (B) Opened siliques (from top to bottom): *cycd1;1*, *mea/MEA cycd1;1/CYCD1;1, mea/MEA cycd1;1*, and *mea cycd1;1* plants with percentage of viable seeds indicated on the right. (C–E) DIC microscopy images of seeds from *mea cycd1;1* plants showing a *mea*-like seed (C), a seed with a WT-looking embryo surrounded by *mea*-looking endosperm (D), and a seed with a giant embryo (E). Arrowhead indicates uncellularized endosperm. Scale bar, 20μm

Expression of translational and transcriptional reporter genes for *CYCD1;1* (*pCYCD1;1::CYCD1;1-GFP-3’UTR* and *pCYCD1;1::NLS-3xVenus-3’UTR*, respectively) showed that *CYCD1;1* expression in WT embryos begins around the late globular/transition stage, with an initial localization restricted to the hypophyseal area (Figures 5A, S4B, and S4C). Afterwards, *CYCD1;1* expression marks the QC, the columella stem cells, and the provascular tissue (Figures S4B and S4C). At late stages of seed development, *CYCD1;1* expression was found in several embryonic cell types but was specifically excluded from the QC (Figure S4B). In the adult plant, we also detected overlapping expression profiles between the translational and transcriptional reporter genes in tissues such as the primary root, lateral roots, and the seed coat (Figures S4B and S4C).

We introduced both translational and transcriptional reporter genes into the *mea/MEA* background to visualize the *CYCD1;1* expression profile in *mea* embryos. However, imaging the translational *pCYCD1;1::CYCD1;1-GFP-3’UTR* fusion protein in a large number of embryos gave inconsistent results, even in the WT, due to its very low signal intensity, most likely caused by the oscillating nature of CYCD1;1 protein levels. Given that *pCYCD1;1::CYCD1;1-GFP-3’UTR* and *pCYCD1;1::NLS-3xVenus-3’UTR* showed the same expression profile in all tissues analyzed (Figures S4B and S4C), we characterized *CYCD1;1* expression in *mea/MEA* seeds using the transcriptional *pCYCD1;1::NLS-3xVenus-3’UTR* reporter gene.

The upregulation of the *CYCD1;1* detected by transcriptomic and ddPCR approaches was further supported by the visualization of a *pCYCD1;1::NLS-3xVenus-3’UTR* marker line, which revealed clear ectopic expression of *CYCD1;1* in the basal part of almond-shaped *mea* embryos around the globular stage (19.3%, n = 119; Figures 5A and S5A–S5C). A nuclear Venus signal, although very weak, was detected in some 4-cell stage embryos of *mea/MEA* plants (21%, n=19, Figures 5A and S5A–S5C), which was not be observed in WT *pCYCD1;1::NLS-3xVenus-3’UTR* plants imaged under the same conditions (Figures 5A, S4A, and S5A–S5C). This indicates that *CYCD1;1* transcription is regulated by MEA in very young embryos soon after fertilization. Later in development, around the early heart stage, 47.6% of *mea/MEA* seeds (n = 21, Figures 5A, and S5A–S5C) exhibited embryos with ectopic expression of *pCYCD1;1::NLS-3xVenus-3’UTR* to different degrees, correlating with the severity of the morphological defects (Figures S4B and S5A), with some embryos expressing *pCYCD1;1::NLS-3xVenus-3’UTR* in almost every cell (Figures 5A and S5A). Notably, the signal was undetectable in the endosperm of these seeds as well as in sibling WT embryos (Figures S4B, S4C, and S5A), identifying *CYCD1;1* as potentially responsible for the embryonic patterning defects we observed in *mea* embryos.

**Figure 6:**
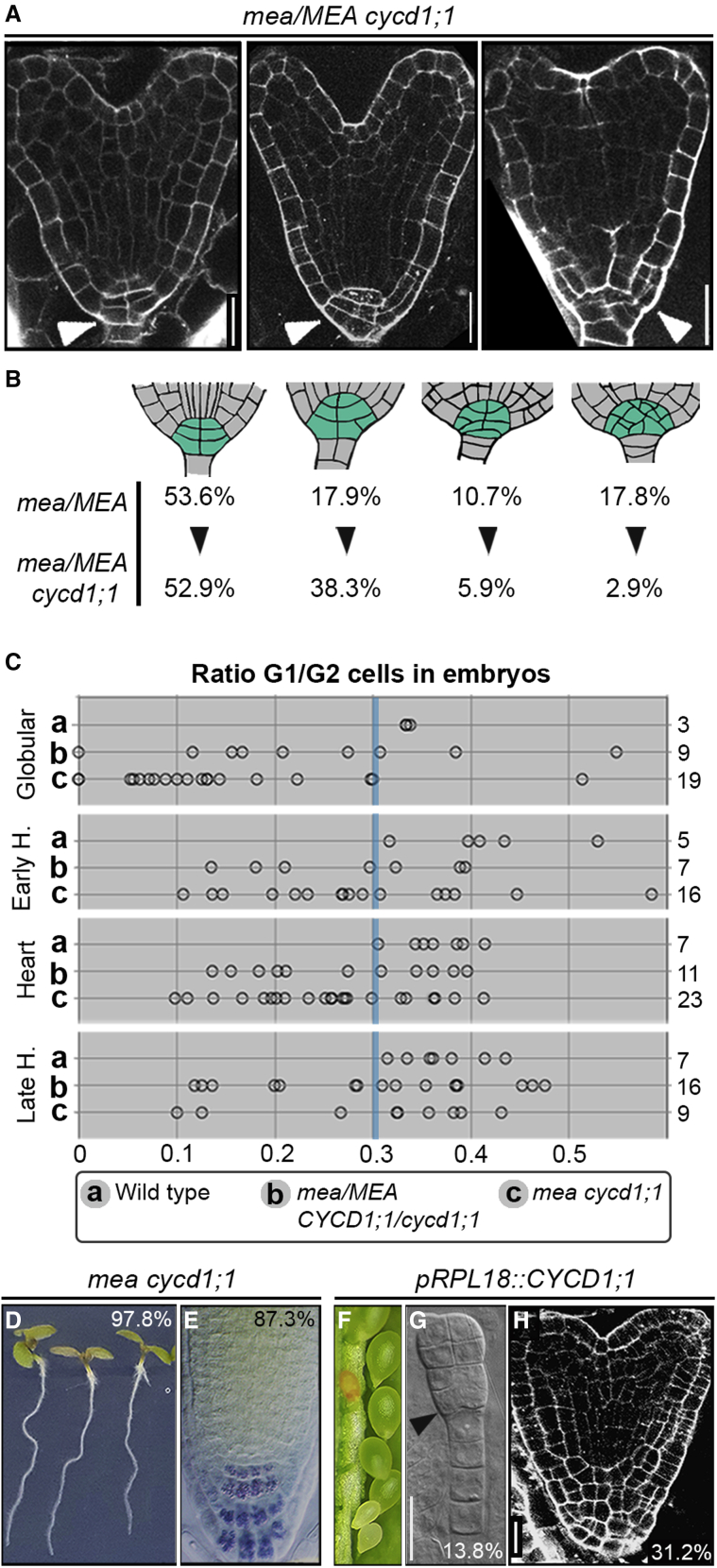
MEA patterns the embryo through regulation of a core cell-cycle component (A) mPS-PI staining of seeds of a *mea/MEA cycd1;1* double mutant plant showing WT-looking embryos (left), embryos with few extra divisions in the columella/QC area (middle), and *mea*-like embryos (right). (B) Schematic representation of cell number and organization in the columella/QC region in *mea/MEA cycd1;1* embryos compared with *mea/MEA*. The number of embryos showing a given range of cells is represented as a percentage. (C) Quantification of the G1/G2 ratio in embryos of WT (A), *mea/MEA cycd1;1/CYCD1;1* (B), and *mea cycd1;1* (C) plants. Values for WT and WT-looking embryos are higher than 0.3 (blue vertical bar). Numbers on the right side refer to the number of embryos imaged. (D and E) Gravitropic response of vertically grown *mea cycd1;1* seedlings (D) and Lugol staining of the primary root tip (E). (F–H) Phenotype of *pRPL18-CYCD1;1* plants showing seed abortion (F), early globular embryo with ectopic cell proliferation at the base (G), and mPS-PI staining of an embryo with excessive and disorganized cell divisions in the columella/QC area. Scale bar, 20μm

To verify whether the ectopic expression of *CYCD1;1* in *mea* embryos is the cause of the *mea* embryonic defects, we crossed *mea/MEA* plants with *cycd1;1* homozygous individuals. In *mea/MEA cycd1;1* double mutants, seed abortion was reduced from the 50% characteristic of *mea/MEA* plants to 35.5% (n = 816; Figure 5B). Analysis of the F3 generation confirmed that the rescued seeds had inherited a maternal *mea* allele, with 8.0% of the progeny of *mea/MEA cycd1;1* plants being homozygous for *mea* (n = 226; Figure 5B). Doubly homozygous *mea cycd1;1* plants exhibited 41.2% viable seeds (n = 1,883) of swollen and rounded appearance, *mea*-like endosperm, and mildly deformed giant embryos (Figures 5C–5E and S6A). Morphological analysis revealed that the embryos in the seeds of *mea/MEA cycd1;1* plants fell into three phenotypic classes (Figure 6A): (1) WT embryos, (2) *mea*-like embryos, and (3) embryos with a significantly reduced number of cells in the columella/QC region as compared with *mea* embryos (Figures 6A, 6B, and S6B–S6E).

Visualization of the triple cell-cycle marker line (PlaCCI, Desvoyes et al., 2020) also confirmed that *mea cycd1;1* embryos developed in a more normally patterned fashion and at a slower pace than *mea* embryos (Figure 6C). Indeed, the rate at which cells progress through the G1 phase was significantly restored in *mea cycd1;1* homozygous embryos in comparison to *mea/MEA* (Figures 4K and 6C) and *mea/MEA CYCD1;1/cycd1;1* individuals (Figure 6C). This effect was particularly evident in embryos from the early heart stage onwards (Figure 6C). Remarkably, 37.5% of early heart stage, 30.4% of heart stage, and 66.7% of late heart stage *mea cycd1;1* embryos exhibited values similar to those of WT embryos at a similar stage (Figure 6C). As a consequence of a less disorganized root meristem and a more regular cell cycle progression, *mea cycd1-1* seedlings showed restoration of the primary root’s gravitropic response (Figures 6D and 6E). These results unequivocally demonstrate that the removal of *CYCD1;1* activity is sufficient to rescue *mea* embryos and leads to a bypass of their growth arrest, even if they are surrounded by abnormal *mea* endosperm.

In agreement with *CYCD1;1* being able to impose abnormal embryonic cell divisions, ectopic expression of *CYCD1;1* in WT embryos induced seed abortion in the range of 5% to 20% (*pRPL18::CYCD1;1*; Figure 6F). The embryos arrested at the late globular stage and exhibited proliferation defects in their basal parts (13.8%, n = 894, Figures 6G, S7A, and S7B), reminiscent of almond-shaped *mea* embryos at a similar stage (Figure 2B). Furthermore, 31.2% of the viable embryos showed aberrant division planes in the root meristem (n = 461, Figure 6H). Remarkably, although the *pRPL18* promoter also drives expression in the endosperm (Yan et al., 2016) (Figure S7C), none of the 18 *pRPL18::CYCD1;1* lines analyzed showed abnormal endosperm proliferation and/or cellularization (Figure S7B). These results show that ectopic expression of *CYCD1;1* is sufficient to phenocopy the patterning defects observed in *mea* embryos, independent of the genotype of the endosperm.

**Figure 7:**
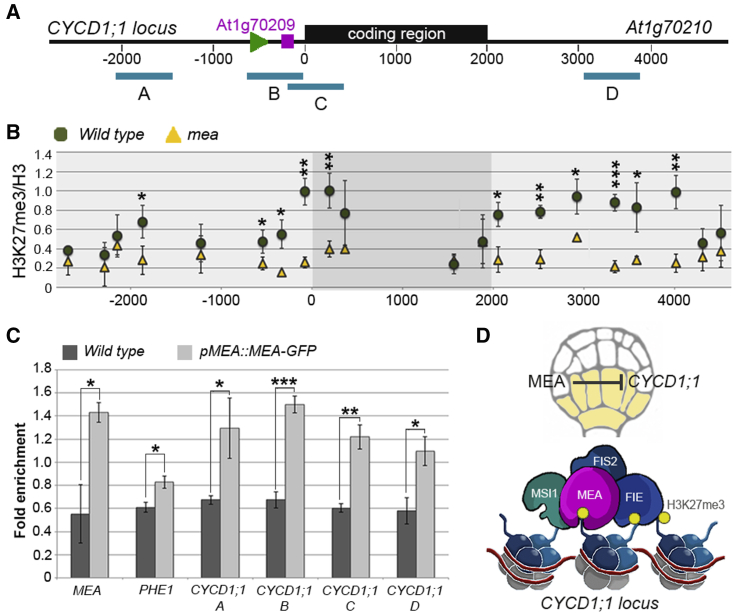
*CYCD1;1* is a direct target of MEA in the embryo (A) Schematic representation of the *CYCD1;1* (*AT1G70210*) locus depicting the coding region (black rectangular box), the position of the *CYCD1;1* transcriptional start site (green arrowhead, position −459), the position of the short *AT1G70209* gene (purple bar, coordinates form −218 to −362), and the regions (A–D) tested in (C). (B) CUT&RUN analysis of H3K27me3 over H3 occupancy at the *CYCD1;1* locus in WT versus *mea* embryos. CUT&RUN was performed in biological triplicate for each genotype. Error bars: standard deviation. p value of a *t* test: ^∗^ < 0.05, ^∗∗^ < 0.01, ^∗∗∗^ < 0.001. (C) Chromatin immunoprecipitation (ChIP) of *pMEA::MEA-GFP* versus the WT demonstrating direct binding of MEA at the *CYCD1;1* locus (regions A–D). *MEA* and *PHE1* are positive controls. Error bars: standard deviation. ChIP was performed in biological triplicate for each genotype. p value of a t test: ^∗^ < 0.01, ^∗∗^ < 0.001, ^∗∗∗^ < 0.0001. (D) Graphical representation of the mechanism underlying *CYCD1;1* regulation in the embryo: direct binding of MEA to the *CYCD1;1* locus at early embryonic stages allows deposition of the repressive H3K27me3 mark, mediating *CYCD1;1* repression and allowing cell proliferation and embryonic patterning to proceed normally.

To determine whether *CYCD1;1* is indeed a direct FIS-PRC2 target gene, we profiled the H3K27me3 levels at the *CYCD1;1* locus (coding region plus 2.5 kb upstream and downstream sequences, Figure 7A) in isolated early globular *mea* and WT embryos by CUT&RUN (Figure 7B). Consistent with the increased expression of *CYCD1;1*, H3K27me3 at the *CYCD1;1* locus was significantly reduced in *mea* embryos compared with the WT. This was particularly pronounced around the transcriptional start site and downstream of the coding region but also in a region about 1.8 kb upstream (Figure 7B). Moreover, we detected direct binding of MEA at the *CYCD1;1* locus in the regions that are highly enriched in H3K27me3 (Regions A–D, Figures 7A–7C), confirming that *CYCD1;1* is a direct target of the MEA-containing PRC2 in the embryo.

Taken together, our results show that *CYCD1;1* is a key target of maternal *MEA* activity and that the deregulation of *CYCD1;1* in *mea* embryos is, to a major extent, responsible for their abnormal development and abortion. Thus, PRC2 directly regulates embryonic patterning and growth by enabling the repression of *CYCD1;1*, a core cell-cycle component.

## Discussion

PcG proteins are central to both animal and plant development (Inoue et al., 2017; Raissig et al., 2013), but through which target genes they exert this control is known for only a few plant developmental processes (Goodrich et al., 1997; Ikeuchi et al., 2015; Köhler et al., 2003b; Lodha et al., 2013). Although mutations affecting FIS-PRC2 cause maternal-effect embryo abortion (Grossniklaus et al., 1998), a direct role of PcG proteins in plant embryogenesis has been dismissed (Bouyer et al., 2011; Kiyosue et al., 1999; Leroy et al., 2007; Scott et al., 1998). We show that, independent of the genotype of the endosperm, *mea* embryos develop severe patterning defects as a result of abnormal cell divisions, clearly demonstrating a direct role of FIS-PRC2 in embryonic patterning. The regulation of embryogenesis by *MEA* is most important at early stages, when cell differentiation and proliferation need to be tightly controlled to establish the proper organization of the body plan and tissue patterning.

The *Arabidopsis* genome encodes at least three PRC2 complexes, each one with a different methyltransferase, i.e., MEA, CLF, and/or SWN. However, MEA is the only catalytic subunit with substantial expression during early embryogenesis (Baroux et al., 2006; Spillane et al., 2007). Our data support a scenario in which MEA represses genes during early embryogenesis via the deposition of H3K27me3 by the FIS-PRC2 at target genes. Because the H3K27me3 mark can be inherited over cell generations, the repressive H3K27me3 mark deposited by MEA might be maintained over cell divisions also at later stages of embryogenesis, when *MEA* expression progressively decays, through the activity of *CLF* and *SWN*, as it was reported for other tissues (Makarevich et al., 2006). Our analyses have shown that *mea* embryos, although not dramatically deformed, show altered tissue patterning and polarity. So far, a similar analysis has not been performed for *clf swn* embryos; therefore, one cannot exclude that also *CLF* and *SWN* regulate embryonic patterning at later stages. However, at a gross level, seeds of *clf swn* double mutants look normal (Chanvivattana et al., 2004), and neither *swn* nor *clf* mutations enhance the seed abortion phenotype of *mea/MEA* plants (Spillane et al., 2007). These findings indicate that the MEA- but not the CLF- and SWN-containing PRC2 is involved in the epigenetic control of early embryogenesis.

Overproliferation in *mea* embryos is caused by de-repression of the core cell-cycle component *CYCD1;1*, which is known to promote the rate and direction of cell divisions (Forzani et al., 2014; Sozzani et al, 2010; Meijer and Murray, 2000). *CYCD1;1* is usually silenced by MEA-mediated H3K27me3 in early WT embryos. *CYCD1;1* is a major direct target of MEA as its overexpression in the WT causes defects reminiscent of those observed in *mea* embryos, and the *cycd1;1* mutant largely suppresses the *mea* phenotypes. However, as suppression of *mea* seed abortion is incomplete, additional MEA target genes may play a minor role in embryonic patterning. The expression of *CYCD1;1* progressively increases during embryogenesis concomitant with the reduction of *MEA* transcript levels (Baroux et al., 2006). This is in support of the idea that *MEA* spatially and temporally establishes the epigenetic landscape to achieve coordinated cell proliferation and differentiation during embryonic growth. It also supports the hypothesis that the H3K27me3 mark is maintained in daughter cells by PRC2. Histone marks such as H3K27me3 can be passively diluted over cell divisions, as previously demonstrated both in animals and plants (Jadav et al., 2020; Sun et al., 2014). This could also be the case at the *CYCD1;1* locus, concomitant with the decrease in MEA activity around the globular embryonic stage, leading to a passive dilution of the repressive H3K27me3 mark over consecutive cell divisions. This passive dilution allows gradual reactivation of FIS-PRC2 target genes at later developmental stages if they are not also targeted by other PRC2s.

A suppression of the *mea* seed abortion phenotype was also observed in the progeny of crosses between *mea/MEA* and *cdka;1/CDKA;1* plants (Nowack et al., 2006, 2007). CDKA;1 is a cyclin-dependent kinase which, by interacting with D-type cyclins, mediates the phosphorylation of the cell cycle master regulator RETINOBLASTOMA-RELATED1 (RBR1), thereby promoting entry and progression into S phase (reviewed in Desvoyes and Gutierrez, 2020). Given that CDKA;1 interacts with CYCD1;1 (Boruc et al., 2010), the increased levels of *CYCD1;1* in *mea* embryos could promote the formation of functional CDKA;1-CYCD1;1 complexes, thereby accelerating cell-cycle progression. Consequently, suppression of *mea* embryo abortion by both the *cdka;1* and *cycd1;1* mutations may be due to the fact that they are part of the same protein complex.

The identification of *CYCD1;1* as a target of MEA suggests that *Arabidopsis* PcG proteins exert a direct control over cell-cycle progression as was reported for animal PcG proteins (Martinez et al., 2006; Iovino et al., 2013; Su et al., 2015; von Schimmelmann et al., 2016; Adhikari and Davie, 2020). So far, only a few MEA target genes have been identified in the context of seed development, with *PHE1* being the best characterized (Köhler et al, 2003b). PHE1 is a MADS-box transcription factor that plays a major role in regulating imprinted genes in the endosperm (Köhler et al., 2003b; Batista et al., 2019). *CYCD1;1* is not only the first PRC2 target gene that is specifically involved in embryogenesis, but also the first factor that directly links PRC2-based gene regulation with the control of cell proliferation in plants. Although mutants disrupting components of FIS-PRC2 are characterized by defects in cell proliferation, none of the characterized target genes provided a link to cell-cycle regulation.

The PRC2 regulatory complex is conserved from animals to plants (Grossniklaus and Paro, 2014) and, thus, arose in their common, unicellular ancestor before the split of the two kingdoms. Interestingly, PRC2 regulates cell proliferation and pattern formation not only in plants as shown here but also in animals (O’Carroll et al., 2001; O’Dor et al., 2006; Oktaba et al., 2008; Pasini et al., 2004), despite the fact that multicellularity evolved independently in these lineages. Similarly, genomic imprinting arose through convergent evolution in plants and mammals but does exert growth control and is partly regulated by PRC2 in both these lineages (Barlow and Bartolomei, 2014; Ferguson-Smith, 2011; Grossniklaus and Paro, 2014; Pires and Grossniklaus, 2014). It is possible that PRC2 had an ancient role in regulating the cell cycle in the common ancestor of animals and plants and that this repressive regulatory module was then exploited as pattern formation evolved in multicellular organisms and again as a placental habit and genomic imprinting arose in seed plants and mammals, respectively (Inoue et al., 2017; Pires and Grossniklaus, 2014). Thus, the regulation of cell proliferation by PRC2 seems to form a robust regulatory module that was independently recruited into various epigenetically controlled processes during the evolution of multicellular organisms.

### Limitations of the study

In this study, we showed that plant embryonic patterning and growth are epigenetically regulated by PRC2 through direct regulation of *CYCD1;1*, encoding a D-type cyclin. To a large extent, introgression of the *cycd1-1* mutation into the *mea*/*MEA* background rescued embryonic defects and allowed many *mea*/*mea* seeds to develop to maturity. However, suppression of the embryo abortion phenotype was not fully penetrant and some *mea* embryos were not rescued or still showed morphological defects. This observation suggests that other yet uncharacterized PRC2 target genes are involved in regulating embryonic development.

We presented the H3K27me3 profile at the *CYCD1;1* locus in embryos through CUT&RUN and direct binding of MEA to *CYCD1;1* using ChIP. These are robust and reliable techniques that can be easily adopted in a molecular biology laboratory. However, to perform analyses similar to the ones we presented here, one has to take into account the time needed to collect sufficient amounts of plant material. In our case, we manually collected about 30,000 embryos to perform CUT&RUN and expression analyses. We collected them over a period of 6 months, performing a few rounds of harvesting each day at a maximal rate of approximately 150 embryos/hour. Thus, performing such assays in rare cell types or tissues requires a considerable commitment.

## STAR★Methods

### Key resources table

**Table:** 

REAGENT or RESOURCE	SOURCE	IDENTIFIER
**Antibodies**		

α-H3K27me3 (rabbit)	Abcam	Catalog # ab192985 RRID: AB_2650559
α-H3 (rabbit)	Abcam	Catalog # ab1791 RRID: AB_302613

**Chemicals**		

Mini Protease EDTA-free Inhibitor Cocktail	Sigma-Aldrich	Catalog # 11873580001
Na_2_HPO_4_	Merck	Catalog # 1.06580.1000
KH_2_PO_4_	Merck	Catalog # 1.04873.1000
Tween-20	Sigma-Aldrich	Catalog # P9416-50ML
HEPES	Sigma-Aldrich	Catalog # H3375.250G
KCl	Sigma-Aldrich	Catalog # 60130-1KG
CaCl_2_	Sigma-Aldrich	Catalog # 21074-1KG
MnCl_2_	Merck	Catalog # 1.05927.0100
Spermidine	Sigma-Aldrich	Catalog # S2626-1G
NaCl	Roth	Catalog # 3957.1
BSA	Sigma-Aldrich	Catalog # A4503-100G
EDTA	HUBERLAB	Catalog # A2937.1000
EGTA	Sigma-Aldrich	Catalog # E4378-25G
Glycogen	Roche	Catalog # 10901393001
Phenol:Chloroform:Isoamyl alcohol 25:24:1	Sigma-Aldrich	Catalog #77617-100ML
Ethanol	Fisher Scientific	Catalog # E/0665DF/17
Formaldehyde	Sigma-Aldrich	Catalog # F1635-500ML
DSG (disuccinimidyl glutarate)	Thermo Scientific	Catalog # 11836794
Hexylene glycol	Sigma-Aldrich	Catalog # 68340-500ML
MgCl_2_^∗^ 6H_2_0	Sigma-Aldrich	Catalog # M2670-500G
DTT (1,4-Dithioerythritol)	Merck	Catalog # 1.24511.0005
SDS (pellets)	Roth	Catalog # CN30.3
Na_2_HPO_4_^∗^ 2H_2_0	Merck	Catalog # 1.06580.1000
NaH_2_PO_4_^∗^ H_2_0	Applichem	Catalog # A1047,1000
Triton X-100	Sigma-Aldrich	Catalog # T8787-100ML
Tris	Roth	Catalog # AE15.3
Acetic acid	Merck	Catalog # 1.00063.1000
Chloral hydrate	Sigma-Aldrich	Catalog # 15307.500G-R
MS salt base	Carolina Biologicals	Catalog # 19-5703
Sucrose	HUBERLAB	Catalog # A2211.1000
Kanamycin sulfate	Applichem	Catalog # A1493,0025
Gentamicin sulfate	Roth	Catalog # 0233.3
Glucose	Sigma-Aldrich	Catalog # G7021-1KG
MES	Roth	Catalog # 6066.2
Phytoagar	Duchefa	Catalog # P1003.1000
Lugol	Sigma-Aldrich	Catalog # 62650
Propidium Iodide	Sigma-Aldrich	Catalog # P4170
Glycerol	Roth	Catalog # 3783.1
CTAB	Sigma-Aldrich	Catalog # H6269-500G
ß-mercaptoethanol	Sigma-Aldrich	Catalog # M7154-100ML
LiCl	Sigma-Aldrich	Catalog # L9650-100G

**Deposited Data**		

RNA-Seq	This paper	ArrayExpress: E-MTAB-9569

**Experimental Models: Organisms/Strains**		

*Arabidopsis thaliana* accession Col-0	Standard accession	N/A
*Arabidopsis thaliana* accession L*er*	Standard accession	N/A
Arabidopsis thaliana mutant *mea-1* (L*er*)	Grossniklaus et al., 1998	N/A
*Arabidopsis thaliana* mutant *mea-2* (L*er*)	Grossniklaus et al., 1998	N/A
*Arabidopsis thaliana pMEA::MEA-GR* in *mea-1/mea-1* (L*er*)	Pires et al., 2016	N/A
*Arabidopsis thaliana DR5V2* (Col-0)	Liao et al., 2015	N/A
*Arabidopsis thaliana pPIN7::PIN7-GFP* (Col-0)	Vieten et al., 2005	NASC ID: N9577
*Arabidopsis thaliana pPLT1::PLT1-YFP* (Col-0)	Galinha et al., 2007	N/A
*Arabidopsis thaliana pBBM::BBM-YFP* (Col-0)	Galinha et al., 2007	N/A
*Arabidopsis thaliana pTMO5::3xGFP* (Col-0)	Schlereth et al., 2010	N/A
*Arabidopsis thaliana pSCR::SCR-GFP* (Ws)	Wysocka-Diller et al., 2000	NASC ID: N3999
*Arabidopsis thaliana pCLV3::GFP-ER pWUS::dsRED-N7* (L*er*)	Gordon et al., 2007	NASC ID: N23895
*Arabidopsis thaliana* mutant *cycd1-1* (GABI_214D10); (Col-0)	Kleinboelting et al., 2012	NASC ID: N420494
*Arabidopsis thaliana PlaCCI* (triple cell-cycle marker line) (Col-0)	Desvoyes et al., 2020	N/A
*Arabidopsis thaliana pCYCD1;1-NLS-3xVenus-3’UTR* (L*er*)	This paper	N/A
*Arabidopsis thaliana pCYCD1;1-CYCD1;1-GFP-3’UTR* (L*er*)	This paper	N/A
*Arabidopsis thaliana pRPL18::CYCD1;1* (L*er*)	This paper	N/A
*Arabidopsis thaliana pRPL18::NLS-3xVenus* (Col-0)	This paper	N/A
*Arabidopsis thaliana pWOX5::dsRED* (Col-0)	This paper	N/A
*Arabidopsis thaliana pMEA::MEA-GFP-3’UTR* in *mea-1/mea-1* (L*er*)	This paper	N/A
*Arabidopsis thaliana kpl* mutant	Ron et al., 2010	N/A
*Arabidopsis thaliana kpl pRPS5A-GFP*	This paper	N/A
*Arabidopsis thaliana mea-1/mea-1 pRPS5A-TagRFP pRPS5a::MEA*	This paper	N/A

**Oligonucleotides**		

All oligonucleotides are listed in Table S2	This paper	N/A
Oligo dT	Invitrogen	Catalog # 18418012

**Recombinant DNA**		

Plasmid *pCYCD1;1-NLS-3xVenus-3’UTR*	This paper	N/A
Plasmid *pRPL18::CYCD1;1*	This paper	N/A
Plasmid *pRPL18::NLS-3xVenus-3’UTR*	This paper	N/A
Plasmid *pCYCD1;1::CYCD1;1-GFP-3’UTR*	This paper	N/A
Plasmid *pMEA::MEA-GFP*	This paper	N/A
Plasmid *pPZP222*	Bencivenga et al., 2016	N/A
Plasmid *pWOX5::dsRED*	This paper	N/A
*pEC50505*	Weber et al., 2011	N/A
Plasmid *pRPS5A-GFP*	This paper	N/A
Plasmid *pRPS5A-TagRFP*	This paper	N/A
Plasmid *pMDC107*	Curtis and Grossniklaus, 2003	N/A
Plasmid *pDONR221*	Invitrogen	N/A
Plasmid *pDONR207*	Invitrogen	N/A
Plasmid destination vector *CZN654*	Richard Immink; Dorus Gadella	N/A
Plasmid *pRPS5a::MEA*	This paper	N/A

**Enzymes and enzyme-containing mixes**		

BsaI-HF v2	NEB	Catalog # R3733S
EcoRV	NEB	Catalog # R0195L
PacI	NEB	Catalog # R0547S
XhoI	NEB	Catalog #R0146M
SmaI	NEB	Catalog #R0141L
SalI	NEB	Catalog #R0138M
AscI	NEB	Catalog # R0558S
SphI	NEB	Catalog # R0182M
T4 DNA Ligase	NEB	Catalog # M0202S
Turbo-Dnase	Ambion	Catalog # AM1907
GoTaq G2 DNA Polymerase	Promega	Catalog # M784B
ExTaq linear polymerase	Takara	Catalog # RR001A
Maxima Reverse Transcriptase	Thermo Fisher	Catalog # EP0741
QX200 ddPCR EVAGREEN	BIORAD	Catalog # 1864034
QX200 ddPCR Supermix for Probes (No dUTP)	BIORAD	Catalog # 1863024
Proteinase K (20mg/ml)	Ambion	Catalog # AM2546
Proteinase K (25mg/ml, from powder)	MP Biomedicals	Catalog # PROTK100
pA-Mnase	Skene et al., 2018	N/A
RNAse A	Qiagen	Catalog # 19101
Q5® Site-Directed Mutagenesis Kit	NEB	Catalog # E0554S
BP clonase II Enzyme mix	Thermo Fisher	Catalog # 11789100
LR clonase II Enzyme mix	Thermo Fisher	Catalog # 11791020
SsoAdvanced Universal SYBR Green Supermix	BIORAD	Catalog # 172-5274

**Kits**		

RNeasy Plant Mini extraction kit	Qiagen	Catalog # 74904
Nucleospin Gel and PCR clean up	Macherey-Nagel	Catalog # 740609.50
NTB buffer	Macherey-Nagel	Catalog # 740595.150
Nucleospin Plasmid kit	Macherey-Nagel	Catalog # 740588.250
Mag-Bind Plant DS DNA kit	VWR/OMEGA Bio-Tek	Catalog # M1130-00
Bio-Mag Plus Concanavalin A coated beads	Polysciences	Catalog # 86057-10
μMACS GFP Isolation Kit (Beads)	Miltenyi Biotec	Catalog # 130-091-125
uMACS GFP-isolation kit (μ Columns)	Miltenyi Biotec	Catalog # 130-042-701
TruSeq RNA Sample Prep Kit v2	Illumina	Catalog # RS-122-2001

**Others**		

Glass beads 1.7-2.1mm diameter	Roth	Catalog # A556-1
50μm diameter size capillary	BioMedical Instruments	Catalog # BM100T-10P
Celltrics 100μm	Sysmex	Catalog # 04-004-2328
8-well Tissue Culture Chambers	SARSTEDT	Catalog # REF94.6190.802
Miracloth	Merck Millipore	Catalog # 475855
1.5 tubes for sonication	Diagenode	Catalog # C30010010

### Resource availability

#### Lead contact

Further information and requests for resources and reagents may be directed to and will be fulfilled by Ueli Grossniklaus (grossnik@botinst.uzh.ch).

#### Materials availability

All new materials generated in this study will be available upon request from Ueli Grossniklaus (grossnik@botinst.uzh.ch).

#### Data and code availability

The RNA-Seq raw data have been deposited at ArrayExpress under accession number E-MTAB-9569.

### Experimental model and subject details

#### Plant material and growth conditions

All plants used were *Arabidopsis thaliana* (L.) Heynh of the Columbia (Col-0) accession, unless indicated otherwise. Seeds were sown on half-strength MS media (1/2 MS salt base [Carolina Biologicals, USA], 1% sucrose, 0.05% MES, 0.8% Phytoagar [Duchefa], pH 5.7 with KOH), stratified for 3-4 days at 4°C in the dark, and then moved to long-day conditions (8h dark at 18°C, 16h light at 22°C, 70% humidity). When showing four true leaves, seedlings were transplanted to soil and grown under long-day conditions in a walk-in growth chamber (8h dark, 16h light, 22°C, 70% humidity). Lines used in this study are: *DR5V2* (Liao et al., 2015), *pPIN7::PIN7-GFP* (Vieten et al., 2005) (NASC ID N9577), *pPLT1::PLT1-YFP* (Galinha et al., 2007), *pBBM::BBM-YFP* (Galinha et al., 2007), *pTMO5::3xGFP* (Schlereth et al., 2010), *pSCR::SCR-GFP* (in the Wassilevskija (Ws) accession; Wysocka-Diller et al., 2000) (NASC ID N3999), *pCLV3::GFP-ER pWUS::dsRED-N7* (in the Landsberg *erecta* (L*er*) accession; Gordon et al., 2007) (NASC ID N23895); *cycd1-1* mutant (GABI_214D10); *pMEA::MEA-GR* (Pires et al., 2016); PlaCCI triple cell-cycle marker line (Desvoyes et al., 2020).

The *mea* alleles *mea-1* and *mea-2* are in the L*er* accession and marked by the kanamycin resistance gene (Grossniklaus et al., 1998). When required, progeny of crosses with *mea* were sown on kanamycin half-strength MS plates to select for *mea/MEA* individuals. The various marker lines were introduced into the *mea/MEA* mutant background by crossing, using *mea/MEA* as pollen donor, and analysis conduced in the F1 generation. To ensure equal contributions of the Col-0/Ws and L*er* backgrounds to the population of seeds analyzed, the seeds developing on the F1 plants were first screened for marker expression and then classified into two groups with normal and aberrant expression patterns, respectively.

### Method details

#### Creation of *kpl-GFP*, *MEArescue-RFP* lines and double pollination

The *pRPS5A-GFP* construct was assembled as follow: the *pRPS5a* promoter was cloned into pDONR207 using Gateway cloning and, subsequently, into the pMDC107 destination vector (Curtis and Grossniklaus, 2003).

The *pRPS5A-TagRFP* was generated by Gateway cloning of the *pRPS5a* promoter into pDONR221 and, subsequently, in destination vector CZN654 (based on pB7WG2 (Karimi et al., 2002) but adapted by Richard Immink with TagRFP, a gift from Dorus Gadella).

Flowers around stage 12 of spontaneous *mea-2* homozygous individuals or not DEX-induced *mea-1 pMEA::MEA-GR* (Pires et al., 2016) individuals were emasculated and pollinated 24h later. The first pollination was a minimal pollination with *kpl*-GFP pollen. The second pollination was done with *MEA-rescue+RFP* pollen 4.5h after the first pollination. This timing was chosen based on the speed at which pollen tubes grow in the pistils under our growth conditions, and evaluated by the rate of synergid rupture in ovules mounted in 7% glucose supplemented with 0.1mg/ml of Propidium Iodide (SIGMA, P4170) and imaged by a Leica SP5 microscope (Argon laser, excitation 488nm). Under our growth conditions, about 50% of ovules displayed synergid rupture 3.5h after pollination. Each set of single and double pollination experiment was performed three to five times, with similar results.

#### Creation of *pWOX5::dsRED* line

The WOX5 promoter fragment (Sarkar et al., 2007) was cloned from the AKS32 plasmid as *Pst*I fragment into the ML939 cloning vector digested with *Pst*I, resulting in pEG126. The dsREDer reporter with the *35S* CaMV terminator sequence was cloned from the ML878 plasmid after *Sph*I digest, blunting with T4 DNA polymerase, and a second digest with *Xho*I, into pEG126, digested with *Sma*I and *Sal*I, resulting in pEG279. The *pWOX5:dsREDer* expression cassette was cloned from pEG279 with *Pac*I and *Asc*I into the binary plasmid ML516, digested with *Pac*I and *Asc*I, resulting in pEG280.

The vectors were introduced into *Agrobacterium tumefaciens* strain GV3101, and wild-type Col-0 plants transformed following the floral dip method (Clough and Bent, 1998). At least 20 independent transgenic lines were generated and examined for expression pattern, using the *pWOX5:GFP* line as control. The line analyzed here is a T4 generation homozygous line with medium/high expression. Ectopic expression observed outside the QC area is a direct consequence of the ability of the dsRED protein to make multimers that can be highly stable. Since the QC cells can divide to replace dead stem cells bordering the QC, sometime dsRED signal can be seen in areas neighboring the QC, in our analyses the upper cells of the suspensor. This ectopic signal observed in suspensor cells was excluded from the comparison between wild-type and aberrant embryos.

#### Clearing

Siliques were fixed o/n in fixative (Ethanol:Acetic Acid 9:1 v/v) at room temperature. The following day, the fixative was replaced with 70% ethanol. Seeds were isolated from the valves and mounted in Hoyer’s solution (Chloral Hydrate:Water:Glycerol 10:2,5:1 w/v/w) and left to clear overnight. Small seeds required only a few hours of clearing. Images were taken with a Leica DM6000B or Zeiss DMR microscope, both equipped with differential interference contrast (DIC) filters and ANDOR 5.5 Neo sCMOS cameras.

#### Lugol staining

Seedlings seven days after germination and grown on vertical plates were incubated for 2min in Lugol solution (Sigma 62650), rinsed in water, mounted in clearing solution (Chloral Hydrate:Water:Glycerol 8:4:1 w/v/w), and imaged immediately. Images were taken with a Zeiss DMR microscope equipped with DIC filters and an ANDOR 5.5 Neo sCMOS camera.

#### Cloning of reporter gene lines

The *pCYCD1;1-NLS-3xVenus-3’UTR* marker line includes the promoter region (5,476 bp upstream of the ATG) and the 3’UTR (4,349 bp downstream of the stop codon) of the *CYCD1;1* locus (*At1g70210*). The promoter and 3’UTR fragment were assembled as Golden Gate module together with the NLS localization signal and 3xVenus in the pEC50505 vector, modified to accept L1-L2 Gateway cassettes (Bencivenga et al., 2016). The resulting pEC50505-*pCYCD1;1-NLS-3xVenus-3’UTR* cassette was recombined through an LR reaction with in the pPZP222 vector.

For the *pRPL18::CYCD1;1* transgenic line, the RPL18 (Yan et al., 2016) promoter was cloned as Golden Gate module upstream of the *CYCD1;1* coding sequence (CDS, no introns) in the Golden Gate acceptor version of pPZP222; as terminator, the *35S* terminator was placed downstream the *CYCD1;1* CDS.

For the *pRPL18::NLS-3xVenus*, the modules for pRPL18, NLS, 3xVenus, and *35S* terminator were assembled in the pPZP222 vector.

The *pCYCD1;1-CYCD1;1-GFP-3’UTR* construct includes the promoter region (5,476 bp upstream of the ATG), the 3’UTR (4,349 bp downstream of the stop codon), and the *CYCD1;1* genomic locus (*At1g70210*) including introns. The three fragments (promoter, gene, and 3’UTR) were assembled as a Golden Gate module together with the GFP module as a C-term fusion, and introduced into the Level 2 Golden Gate vector pEC50505 (Weber et al., 2011), harboring the kanamycin resistance marker. Where necessary, site-specific mutagenesis was used to remove endogenous *Bsa*I sites.

For the *pMEA::MEA-GFP-3’UTR* complementation construct, the *MEA* locus (4,526bp upstream the ATG, 3’UTR of 1,276bp downstream, and the CDS including introns) was fragmented and amplified in eight modules in order to mutagenize the *Bsa*I endogenous sites. The GFP was introduced between fragment four and five as an in-frame fusion within the seventh exon. *pMEA::MEA-GFP-3’UTR* was introduced into the pEC50505 vector (Weber et al., 2011), modified to accept L1-L2 Gateway cassettes (Bencivenga et al., 2016). The resulting pEC50505*-pMEA::MEA-GFP-3’UTR* cassette was recombined through an LR reaction with the pPZP222 vector.

All vectors were introduced into *Agrobacterium tumefaciens* strain GV3101, and wild-type L*er* (*CYCD1;1* marker lines) or *mea-1/MEA* plants (*pMEA::MEA-GFP-3’UTR* complementation construct) were transformed following the floral dip method (Clough and Bent, 1998). The *pCYCD1;1-NLS-3xVenus-3’UTR* and *pCYCD1;1-CYCD1;1-GFP-3’UTR* transgenes were then introduced from a selected line into the *mea-2/MEA* background by crossing. For *pMEA::MEA-GFP-3’UTR*, line #18, which showed full complementation of the *mea* seed abortion phenotype and harbored a single transgene copy, was made homozygous.

Primers are listed in the Extended Table S2.

#### Droplet Digital PCR (ddPCR)

Genotyping of single seeds: individual seeds were removed from the fruit, deposited in a 1.5ml Eppendorf tube, and grinded with a blue plastic pestle. DNA was extracted with the Mag-Bind Plant DS DNA kit (OMEGA Bio-Tek) following the manufacturer’s instructions. Elution was done with water into a 1.5ml low-binding Eppendorf tube. The DNA samples were then concentrated in a Speedvac to 20μl final volume. Total DNA was digested for 30min at 37°C with *Eco*RV, and then pre-amplified with specific primers for GFP (GFP-fw + GFP-rev), RFP (RFP-fw + RFP-rev), and internal control (Control-fw + Control-rev) genes together in the same reaction tube, using the ExTaq linear polymerase (Takara, RR001A) as follows: DNA 3μl, 40nM of each primer, 0.6 units Extaq in 1x buffer containing 1.5mM MgCl_2_ with the following PCR protocol: 98°C x 3min, [98°C x 10sec; 59°C x 20sec; 72°C x 20sec]x15 cycles. For ddPCR, 5μl of pre-amplified DNA were used in duplex assays GFP/Control and RFP/Control. Assay conditions: 500nM primers, 200nM probes, and QX200 ddPCR Probe no dUTPS Supermix (BioRad). The PCR protocol was the manufacturer’s recommendation for Probe assays (95°C: 10min, 94°C 30s, Ramp 2.5°C/s, 60°C 1min, Ramp 2.5°C/s[40 cycles], 98°C 10min, 4°C until further process). Fluorescence was detected with the QX200 droplet digital reader (Bio-Rad), and analyzed with the provided Quanta Soft version 1.7 software. Presence of each gene was calculated relative to the endogenous control. The sum was given as 100% and the ratio of each gene was calculated as % relative to the total.

Expression analysis of *CYCD1;1* on isolated embryos: seeds were removed from siliques, placed in a 1.5ml Eppendorf tube containing PBS1X, and gently pressed with a blue plastic pestle with up-and-down movements to release the embryos. Collection time did not exceed 15min. The sample was then passed through a 100μm pore-size cell strainer (CellTrics) to remove excess of debris. The flow-through, containing the embryos, was collected in a small plastic rectangular box with low walls (we used the lid of the 8-well Tissue Culture Chambers REF94.6190.802, SARSTEDT). Embryos were collected with a 50μm diameter size capillary (ES-blastocyst injection pipettes, BioMedical Instruments, BM100T-10P) and an oil micromanipulator (CellTram Vario, Eppendorf), mounted on a Leica SP2 inverted microscope. Embryos were collected in maximum 1h shifts and washed thoroughly in fresh PBS1X. The drop of PBS1X buffer containing the embryos was then ejected directly from the capillary onto a piece of parafilm to create a round-shaped droplet. The parafilm was then placed for 2min at -70°C to let the droplet freeze. Frozen droplets were collected in a 1.5ml low-binding Eppendorf tube and stored at -70°C until the extraction. For our experiment, a total of 1000 embryos around the early globular stage (with and without suspensor) were collected per replicate from wild-type and *mea* plants, and ddPCR was performed on biological triplicates (total of 3000 embryos per genotype). For RNA extraction, 4-6 glass beads (1.7-2.1mm diameter, ROTH A556-1) were added to each tube containing the frozen droplets with the embryos, frozen in liquid nitrogen, and grinded 3-4 times with a single-tube tissue grinder (Silamat S6). The RNA extraction was done with the Qiagen RNeasy Plant Mini extraction kit, and subsequently treated with Turbo-Dnase (Ambion) following the manufacturer’s protocol. cDNA synthesis was performed using Maxima Reverse Transcriptase (Invitrogen) and OligodT (Invitrogen) following the manufacturer’s protocol. 5μl of a 1:2 dilution of cDNA were then used for ddPCR assays of *CYCD1;1/UBI21*, with 100nM final concentration of each primer, in a total reaction volume of 25μl, 20 of which were used to generate droplets in 1X Master mix EVAGREEN (BIORAD). PCR conditions were according to manufacturer’s recommendation for EVAGREEN.

Primers are listed in the Table S2.

#### RNA-Seq

The following tissue was harvested for the three different stages: (1) ovaries two days after emasculation (style and stigma were removed), (2) developing seeds 1-2 days after pollination (dissected from siliques), and (3) developing seeds 4 days after pollination (dissected from siliques). Three independent biological replicates were generated for each tissue/genotype combination. For each replicate, the isolated tissues were frozen in liquid nitrogen, ground to a fine powder using a pestle, and incubated for 10min in 450 μl of a solution containing 2% CTAB, 100 mM Tris-HCl pH 8.0, 25 mM EDTA, 2M NaCl, and 2% ß-mercaptoethanol. This suspension was then mixed with ice-cold chloroform and centrifuged 15min at 16000 g. The upper phase was collected and mixed with 150μl of a 8M LiCl solution, incubated at -20°C for 1 h, and centrifuged 30min at 16000 g. The RNA pellet was washed with 70% ethanol and resuspended in 30μl RNAse-free water and quantified using the Qubit. The Turbo DNA free kit (Ambion AM1907) was used to remove DNA. Total RNA samples were polyA-enriched and reverse-transcribed into double stranded cDNA.

Sequencing libraries were generated using the TruSeq RNA Sample Prep Kit v2 (Illumina). Libraries were normalized and pooled using TruSeq index adapters and sequenced using single reads in a Illumina HiSeq 2000 sequencer at the Functional Genomics Centre Zurich. Low quality read ends were clipped and reads were mapped to the TAIR reference genome with TopHat. Differential gene expression was performed using DEseq2. For the differential gene expression analysis between wild-type and *mea* samples, only genes for which more than 4 counts per million were present in most samples were analyzed. After dispersion estimates were obtained, a negative binomial model was fitted and differential expression was tested using a quasi-likelihood F-test, as implemented in EdgeR (using a p-value of 0.01)

#### Confocal imaging, mPS-PI, and PI staining

Confocal analyses were performed using a Leica SP5 confocal microscope. GFP: Argon laser, excitation 488; YFP: Argon laser, excitation 514nm; CFP: argon laser, excitation 456nm; RFP: argon laser excitation 558nm; PI: Argon laser, excitation 488.

mPS-PI of seeds: seeds at different developmental stages were isolated from siliques and treated as in Truernit et al., 2008.

PI staining of primary roots: seedlings were incubated for 10min in propidium iodide (PI) solution at a concentration of 10μ g/ml (Sigma P4170), mounted in 30% glycerol, and imaged with a Leica SP5.

For confocal analysis of *pCYCD1;1::NLS-3XVenus-3’UTR* and cell fate markers in WT and *mea/MEA* plants, each marker line was crossed to *mea/MEA* and the F1 generation analyzed. Embryos were exserted from the seed by gentle pressure and immediately imaged. Each embryo was imaged in a single focal plane, using the suspensor and the QC area as reference point. Embryonic stages were classified according to ten Hove et al., 2015.

#### CUT&RUN

Embryos for CUT&RUN were isolated as described above for the expression analysis of *CYCD1;1*, with the exception that the collection buffer PBS1X was supplemented with the Mini Protease EDTA-free Inhibitor Cocktail (Roche). A total of 3500 embryos were collected around the globular stage per replicate per genotype (wild-type and *mea*). CUT&RUN has been performed in triplicate following the protocols reported by Zheng and Gehring (2019) and Skene et al. (2018), with some minor modifications. Differently from Zheng and Gehring (2019), we included Tween-20 in the binding and washing buffers as in Skene et al. (2018). Antibodies used were: anti-H3K27me3 (Abcam, Catalog#192985) and anti-H3 (Abcam, catalog# 1791). pA-Mnase and the Spike-in DNA were a kind gift of Steve Henikoff. The final pellet of DNA was resuspended in 50μl of TE1X. Real-Time PCR was performed on a BIORAD CFX384 machine, in technical triplicates, on 384-well plates (LightCycler 480 white plates and sealing foils, ROCHE), using 0.7μl of DNA per replicate and the SsoAdvanced Universal SYBR Green Supermix (BIORAD). Results are presented as H3K27me3 enrichment over H3 occupancy. Statistical analyses are based on a *t*-tests. Primers are listed in the Extended Table S2.

#### Chromatin immunoprecipitation (ChIP)

Siliques at 1-4 days after pollination were collected in rounds of maximum 1h at room temperature, submerged in PBS1X supplemented with 1mM PMSF, 2% formaldeyde, and 1μM DSG, and vacuum infiltrated for 20min. Fixation was performed according to ChIP protocols used for proteins that do not directly bind DNA (Serrano-Mislata et al., 2017). Cross-linking was stopped by adding glycine to a final concentration of 0.1M, and samples were incubated for a further 10min. Then, the tissue was rinsed 3 times with water, dried on absorbent paper, frozen in liquid nitrogen, and stored at -80°C. A total of about 45g of siliques was harvested and fixed in order to obtain approximately 15g per replicate. As a negative control, wild-type (WT) plants were used and the tissue treated as above. The ChIP protocol of Schiessl et al. (2014), was followed with some minor modifications. Briefly, the tissue was ground to a fine powder in liquid nitrogen with mortar and pestle, then about 5g of powder were dissolved in 25ml of buffer M1 (10mM NAPI pH 7.0, 0.1M NaCl, hexylene glycol 1M, 10mM β-mercaptoethanol, protease inhibitor cocktail). Therefore, each replicate of about 15g was divided into three 50ml Falcon tubes. Samples were incubated on ice until the powder dissolved, passed through two Miracloth layers, and centrifuged for 20min at 1000g at 4°C. The pellet was gently resuspended in 15ml of buffer M2 (10mM NAPI pH 7, 0.1M NaCl, 1M hexylene glycol, 10mM β-mercaptoethanol, 10mM MgCl_2_, 0.5% Triton X-100, protease inhibitor cocktail), incubated on ice for 15min, and centrifuged for 10min at 1000g at 4°C. The pellet was washed once with 10ml of buffer M3 (10mM NAPI pH7, 0,1M NaCl, 10mM β-mercaptoethanol, protease inhibitor cocktail), and centrifuged for 10min at 1000g at 4°C. The nuclear pellet was resuspended in 1.5ml of Sonication Buffer (50mM HEPES pH 7.5, 150mM NaCl, 5mM MgCl_2_, 1% Triton X-100, protease inhibitor cocktail) and divided between two 1.5ml sonication tubes (i.e., 6 tubes per replicate). Sonication was performed in a Bioruptor bath sonicator (Diagenode) at maximal power for 2 times 5 cycles of 30’’ON/30’’OFF, with a 2min interval between the two rounds to cool down the sample on ice. The samples were then centrifuged for 15min at 14000g at 4°C. The supernatant was transferred to two 5ml Eppendorf tubes (labeled A and B) by combining three 1.5ml tubes belonging to the same replicate. At this point, the sample set consisted of twelve 5ml Eppendorf tubes: sample GFP1A+GFP1B, sample GFP2A+GFP2B, sample GFP3A+GFP3B, sample WT1A+WT1B, sample WT2A+WT2B, and sample WT3A+WT3B. 50μl from each A and B tube belonging to the same replicate were removed and combined together to form a 100μl INPUT sample and stored on ice. Then, to each 5ml Eppendorf tube, an equal volume of Immuno Precipitation+Blocking (IP) buffer was added (50mM HEPES pH 7.5, 150mM NaCl, 5mM MgCl_2_, 1% Triton X-100, 1mg/ml BSA, protease inhibitor cocktail), followed by 30μl of μMACS GFP microbeads (Miltenyi Biotec). Samples were then incubated while rotating for 2h at 4°C. Then, the samples were passed through μ-columns (Miltenyi Biotec) mounted on the magnetic μMACS stand (Miltenyi Biotec), combining A and B tubes of the same replicate on the same μ-column. After all samples passed through the columns, each column was washed as follows: two times 400μl of WASH buffer (WB, 50mM HEPES pH 7.5, 150mM NaCl, 5mM MgCl_2_, 1% Triton X-100), two times 200μl of WB buffer, and two times 200 μl of TE1X buffer. Then, 50 μl of hot (90°C) Elution Buffer (50mM Tris-HCl pH8, 10mM EDTA, 50mM DTT, 1% SDS) was added to each column, and the flowthrough collected in a clean 1.5ml tube. The elution was repeated once more to obtain a final volume of 100μl per sample, and labeled as IP sample. At this point, to each sample, including the INPUT samples, 100 μl of TE1X and 9μl of Proteinase K (25mg/ml) were added and the samples were incubated o/n at 37°C in a thermoblock. The next morning, 9μl of Proteinase K (25mg/ml) were added to each tube, and the samples were incubated at 65°C for another 8 h to revert the crosslinks. After that, DNA was purified using the Macherey-Nagel Gel and PCR purification kit following the manufacturer’s instructions. As the IP samples contain SDS, we used 5 volumes of NBT buffer instead of NBI (as recommended in the Macherey-Nagel manual), by using one column per sample and loading it multiple times when necessary. Each column was eluted with 50 μl of TE1X. Real-Time PCR was performed on a BIORAD CFX384 machine, in technical triplicates, on 384-well plates (LightCycler 480 white plates and sealing foils, Roche), using 1μl of DNA per replicate and the SsoAdvanced Universal SYBR Green Supermix (Biorad). As housekeeping gene, we used the *Mlu*-like transposon. Positive controls for *MEA* were promoter regions of *MEA* (Baroux et al., 2006) and *PHE1* (Kohler et al., 2003b). Results are presented as fold enrichment. Statistical analyses are based on a *t*-test. Primers are listed in the Extended Table S2.

## Supplemental information

Document S1. Figures S1–S7 and Table S2

Table S1. Transcriptomic dataset of wild-type versus mea mutant ovary/seeds, related to Figure 4 Table S1 contains the following: Upregulated and downregulated genes in *mea* versus WT RNA-seq of ovary tissue. Upregulated and downregulated genes in *mea* versus WT RNA-seq of 1-2DAP seeds. Upregulated and downregulated genes in *mea* versus WT RNA-seq of 4DAP seeds. Overlap 4DAP-specific dataset with INTACT dataset of 16C, EG, and LG globular embryos. Word cloud of the unique upregulated genes in all datasets combined. Word cloud of the unique downregulated genes in all datasets combined. Overlap of upregulated and downregulated genes with H3K27me3 targets in the endosperm (dataset from Moreno-Romero et al., 2016). Overlap of upregulated and downregulated genes with upregulated and downregulated genes in *msi1* mutant seeds (dataset from Tiwari et al., 2010). Overlap of upregulated and downregulated genes with direct targets of SWN and CLF (dataset from Shu et al., 2019)

Document S2. Article plus supplemental information
