# Supplementary material for: The Polycomb group protein MEDEA controls cell proliferation and embryonic patterning in Arabidopsis
Source: Dev Cell. 2021 Jul 12;56(13):1945–1960.e7. doi: 10.1016/j.devcel.2021.06.004 (PMC8279741; doi:10.1016/j.devcel.2021.06.004)
Supplement: Document S1. Figures S1–S7 and Table S2 [file mmc1.pdf]

Developmental Cell, Volume *56*

Supplemental information

The *Polycomb* group protein MEDEA controls cell proliferation and embryonic patterning in *Arabidopsis*

Sara Simonini, Marian Bemer, Stefano Bencivenga, Valeria Gagliardini, Nuno D. Pires, Bénédicte Desvoyes, Eric van der Graaff, Crisanto Gutierrez, and Ueli Grossniklaus

Figure S1. Related to Figure 1.

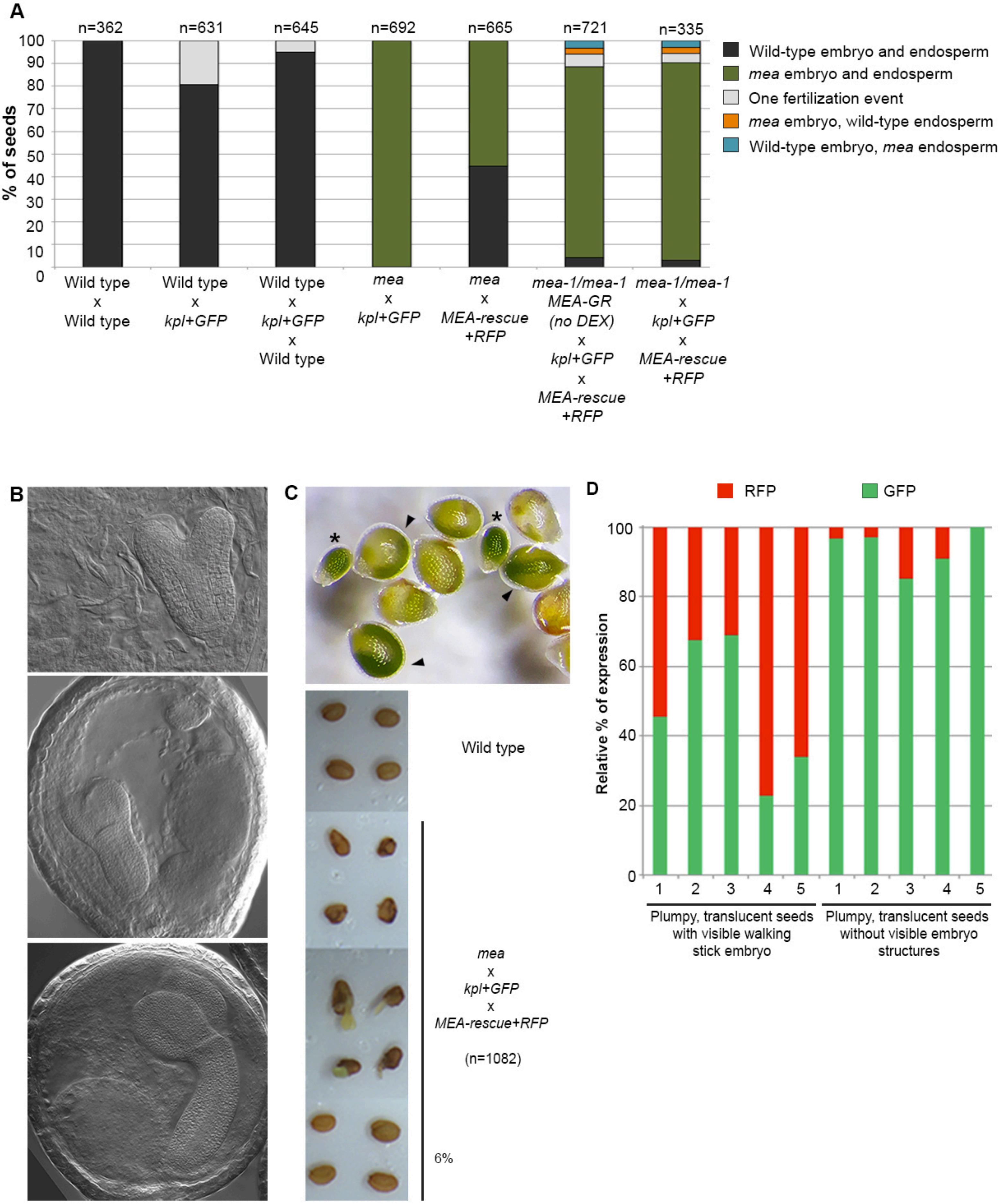

Figure S1: Rescue of mutant *mea* embryos and genotyping of rescued and non-rescued *mea* seeds, related to Figure 1.

A. Quantification of phenotypes observed in the single and double crosses.

B. Images of cleared seeds derived from the double pollination of *mea* pistils. Wild-type embryo is surrounded by uncellularized endosperm.

C. Appearance of developing seeds (top) and dried seeds (bottom) in double pollination of *mea* pistils. The developing embryo (arrow head) is visible in big and swollen seeds, a phenotype that is characteristic of *mea* seeds. Small seeds are the ones derived from full rescue by the *MEA-rescue+RFP* pollen (asterisk).

D. ddPCR analyses for presence of *GFP/RFP* transgenes in single seeds derived from the double pollination of *mea* pistils.

Figure S2. Related to Figure 2.

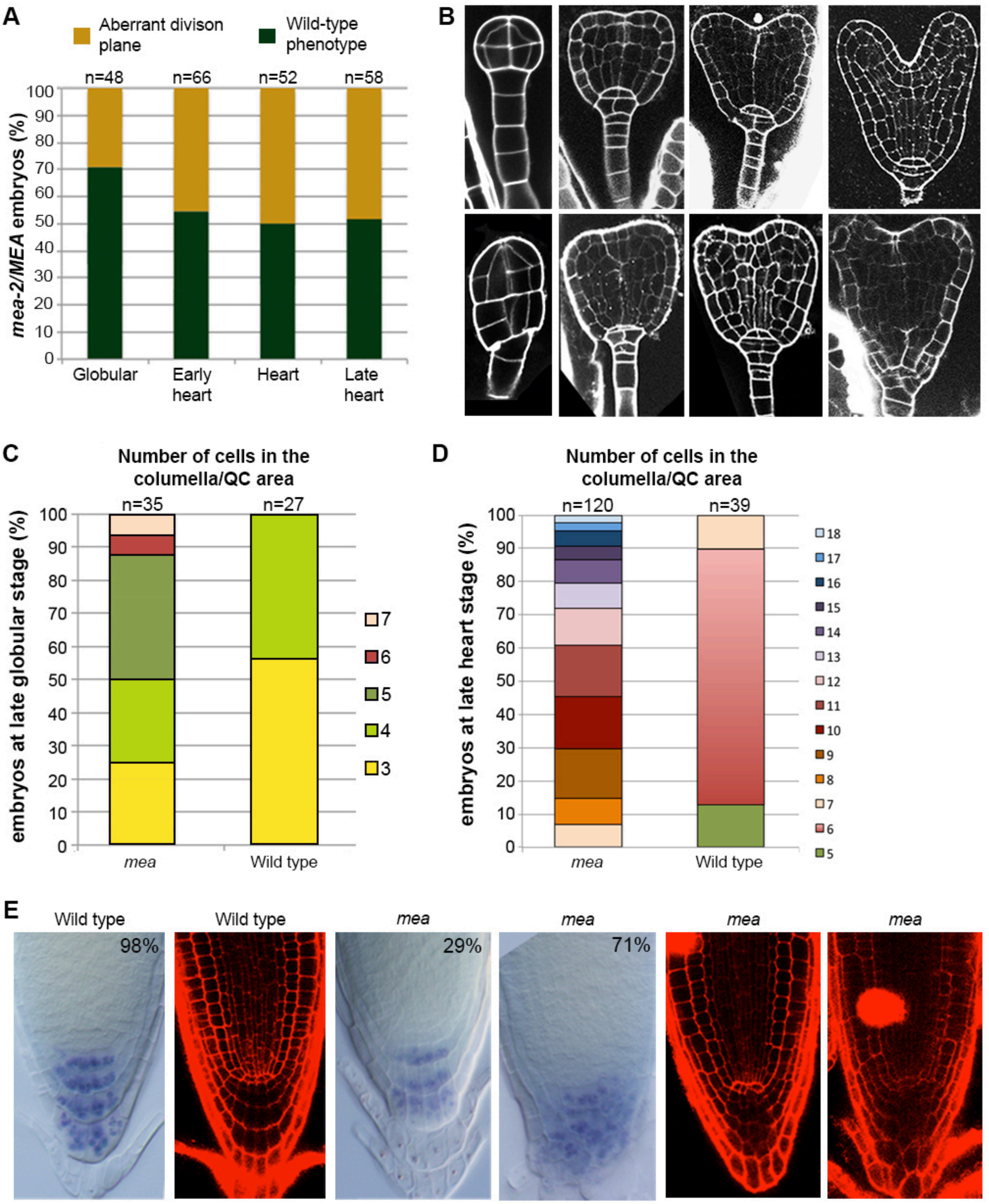

Figure S2: Morphological defects in *mea* embryos from early globular to late heart stages and in the QC-columella area of the primary root of *mea* seedlings, related to Figure 2.

A. Quantification of the number of embryos showing aberrant division planes in *mea/MEA* heterozygous offspring.

B. mPS-PI staining of *mea/MEA* seeds, showing examples picture of embryos from globular (left) to late heart (right) stages.

C. Quantification of the number of cells in the columella/QC area in embryos at late globular stage showing a wild-type phenotype or aberrant divisions.

D. Quantification of the number of cells in the columella/QC area in embryos at late heart stage showing a wild-type or a *mea* phenotype.

E. Lugol staining and Propidium Iodide staining of root tip of wild-type and *mea* homozygous seedlings. The percentage shows the frequency of the phenotype observed in the imaged seedlings (n=14).

Figure S3. Related to Figure 3

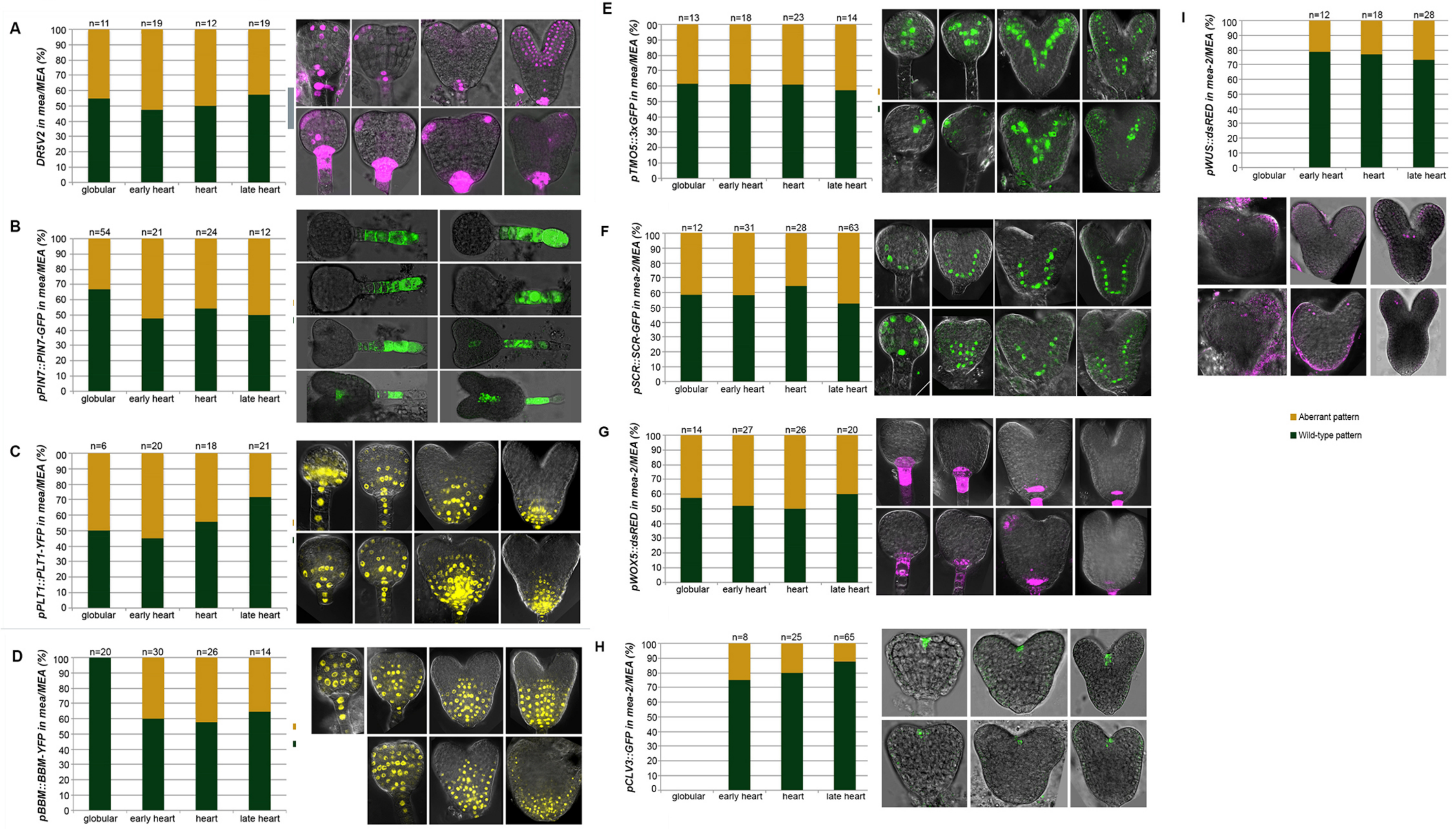

Figure S3. Expression patterns of cell fate markers and their quantification in seeds of *mea/MEA* plants, related to Figure 3.

For all markers, histograms of the quantification are shown on the left and pictures of representative embryos on the right; pictures show wild-type expression patterns in the top row and aberrant ones in the bottom row for all markers, except for (b) where wild-type and aberrant expression patters are shown in the left and right panels, respectively.

A. Quantification of aberrant versus wild-type expression patterns in *mea/MEA DR5V2* seeds.

B. Quantification of aberrant versus wild-type expression patterns in *mea/MEA pPIN7::PIN7-GFP* seeds.

C. Quantification of aberrant versus wild-type expression patterns in *mea/MEA pPLT1::PLT1-YFP* seeds.

D. Quantification of aberrant versus wild-type expression patterns in *mea MEA pBBM::BBM-YFP* seeds.

E. Quantification of aberrant versus wild-type expression pattern in *mea/MEA pTMO5::3xGFP* seeds.

F. Quantification of aberrant versus wild-type expression patterns in *mea/MEA pSCR::SCR-GFP* seeds.

G. Quantification of aberrant versus wild-type expression patterns in *mea/MEA pWOX5::dsRED* seeds.

H. Quantification of aberrant versus wild-type expression patterns in *mea/MEA pCLV3::GFP* seeds.

I. Quantification of aberrant versus wild-type expression patterns in *mea/MEA pWUS::dsRED* seeds.

Figure S4. Related to Figure 5.

A

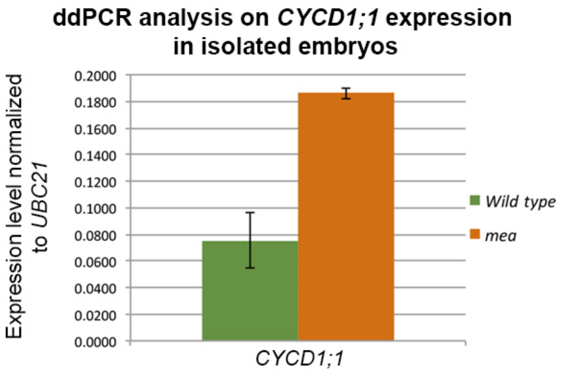

Figure S4: *CYCD1;1* expression in *mea* embryos and confocal analysis of *pCYCD1;1::NLS-3XVenus-3'UTR* in and *pCYCD1;1::CYCD1;1-GFP-3'UTR* marker lines in wild-type plants, related to Figure 5.

A. ddPCR analysis of *CYCD1;1* transcript level in wild-type versus *mea* embryos around early globular stage.

B. Confocal images of *pCYCD1;1::NLS-3xVenus-3'UTR* marker line showing its wild-type expression pattern in embryos from the globular to mature stages, a magnification of the root tip showing no expression in the QC, a seed showing expression only in the sporophytic tissues, the vasculature of the primary root, and two emerging lateral roots.

C. Confocal images of the *pCYCD1;1::CYCD1;1-GFP-3'UTR* reporter gene showing its wild-type expression pattern in embryos from the globular to heart stages, seed coat, vasculature of the primary root, and two emerging lateral roots.

B *pCYCD1;1::NLS-3xVenus-3'UTR (wild-type expression pattern)*

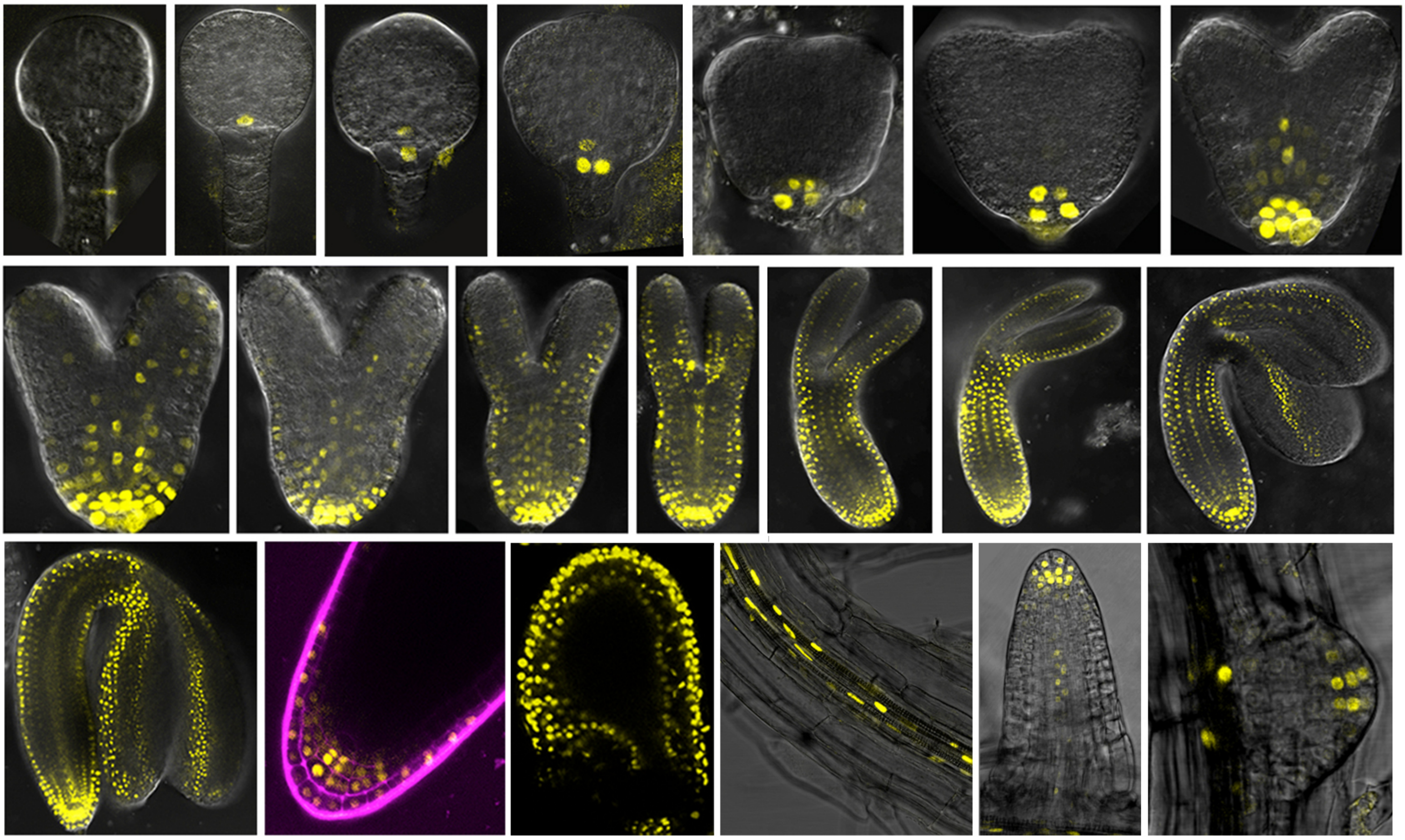

C *pCYCD1;1::CYCD1;1-GFP-3'UTR (wild-type expression pattern)*

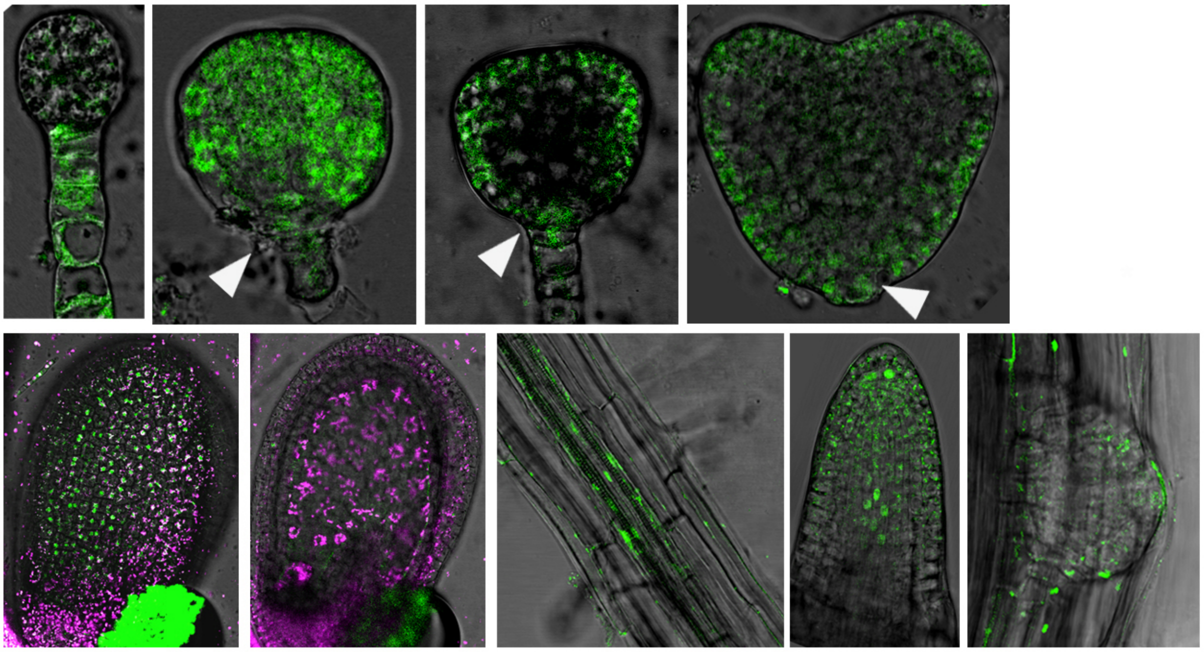

Figure S5. Related to Figure 5.

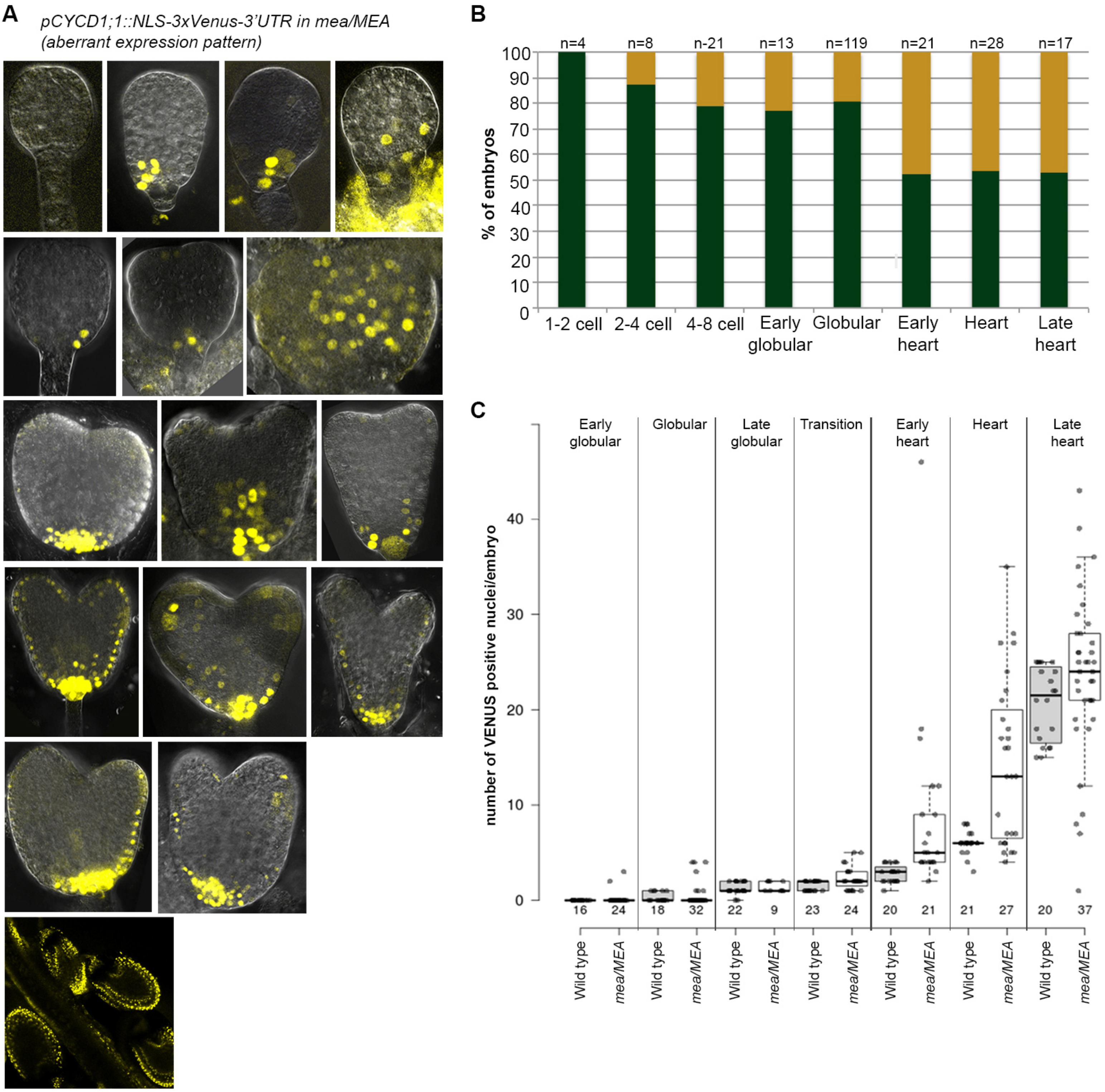

Figure S5: Expression pattern of *pCYCD1;1::NLS-3XVenus-3'UTR* and its quantification in embryos of *mea/MEA* plants at different developmental stages, related to Figure 5.

A. Confocal images of *pCYCD1;1::NLS-3xVenus-3'UTR* marker line showing aberrant expression in *mea/MEA* seeds, in embryos from the globular to late heart stages. In *mea/MEA* seeds, no expression of the marker is detected in the endosperm.

B. Quantification of the percentage of embryos showing an aberrant expression pattern of the *pCYCD1;1::NLS-3xVenus-3'UTR* reporter gene in the *mea/MEA* background.

C. Quantification of the number of nuclei expressing the *pCYCD1;1::NLS-3xVenus-3'UTR* reporter gene in embryos developing from wild-type and *mea/MEA* plants. Embryonic stages from the early globular to the late heart stage are shown.

Figure S6. Related to Figure 5.

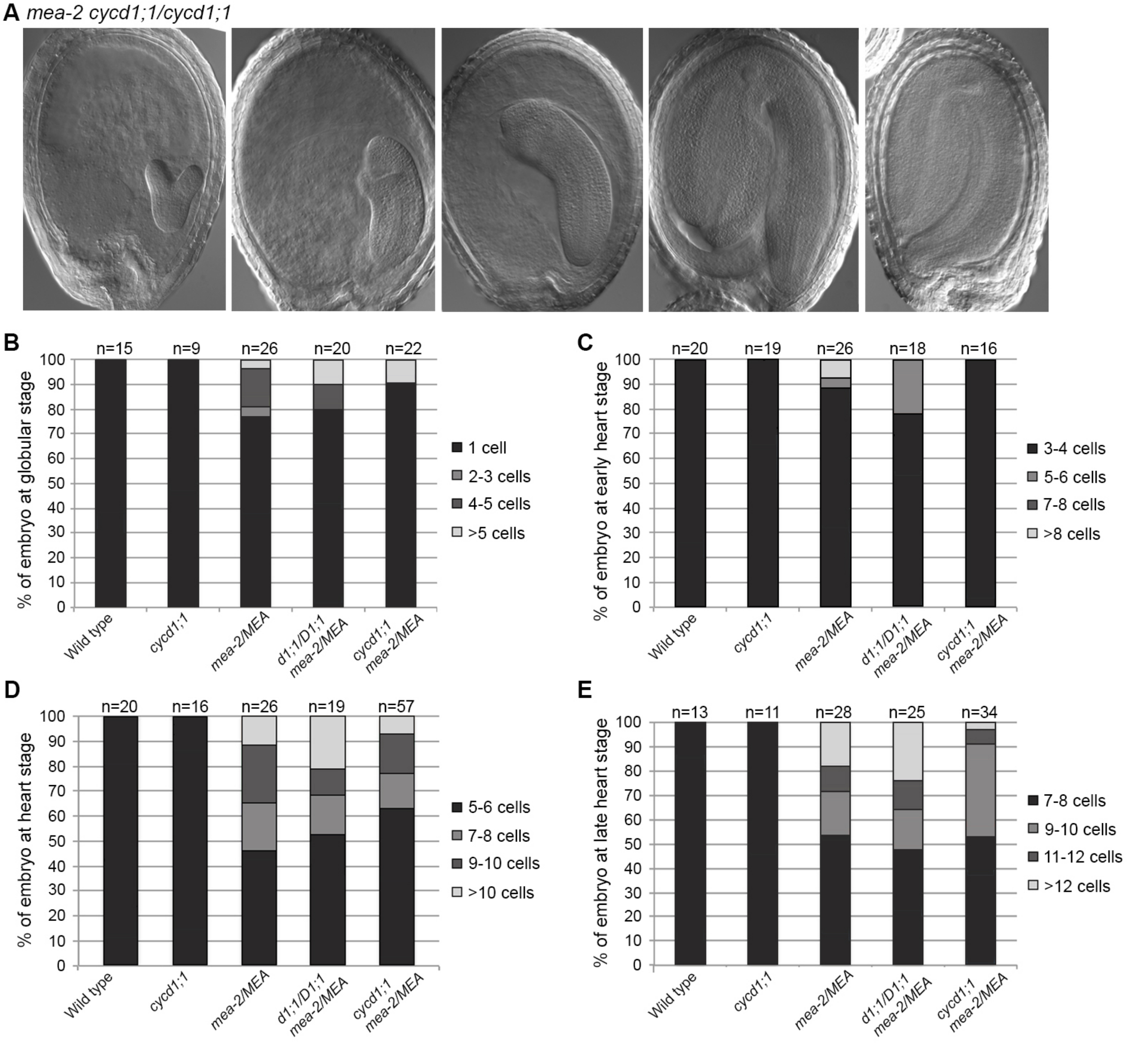

Figure S6. Characterization of *mea cycd1;1* seeds and embryos from globular to late heart stages, related to Figure 5.
A. DIC microscopy analysis of *mea cycd1;1* seeds, showing enlarged embryos surrounded by defective endosperm.
B-E Quantification of the number of cells in the columella/QC area in embryos at globular stage (b), early heart stage (c), heart stage (d), and late heart stage (e), of wild-type,*cycd1;1* mutant, *mea/MEA* mutant, *cycd1;1/CYCD1;1 mea/MEA* double heterozygous mutant, and *cycd1;1 mea/MEA* mutant.

Figure S7. Related to Figure 6.

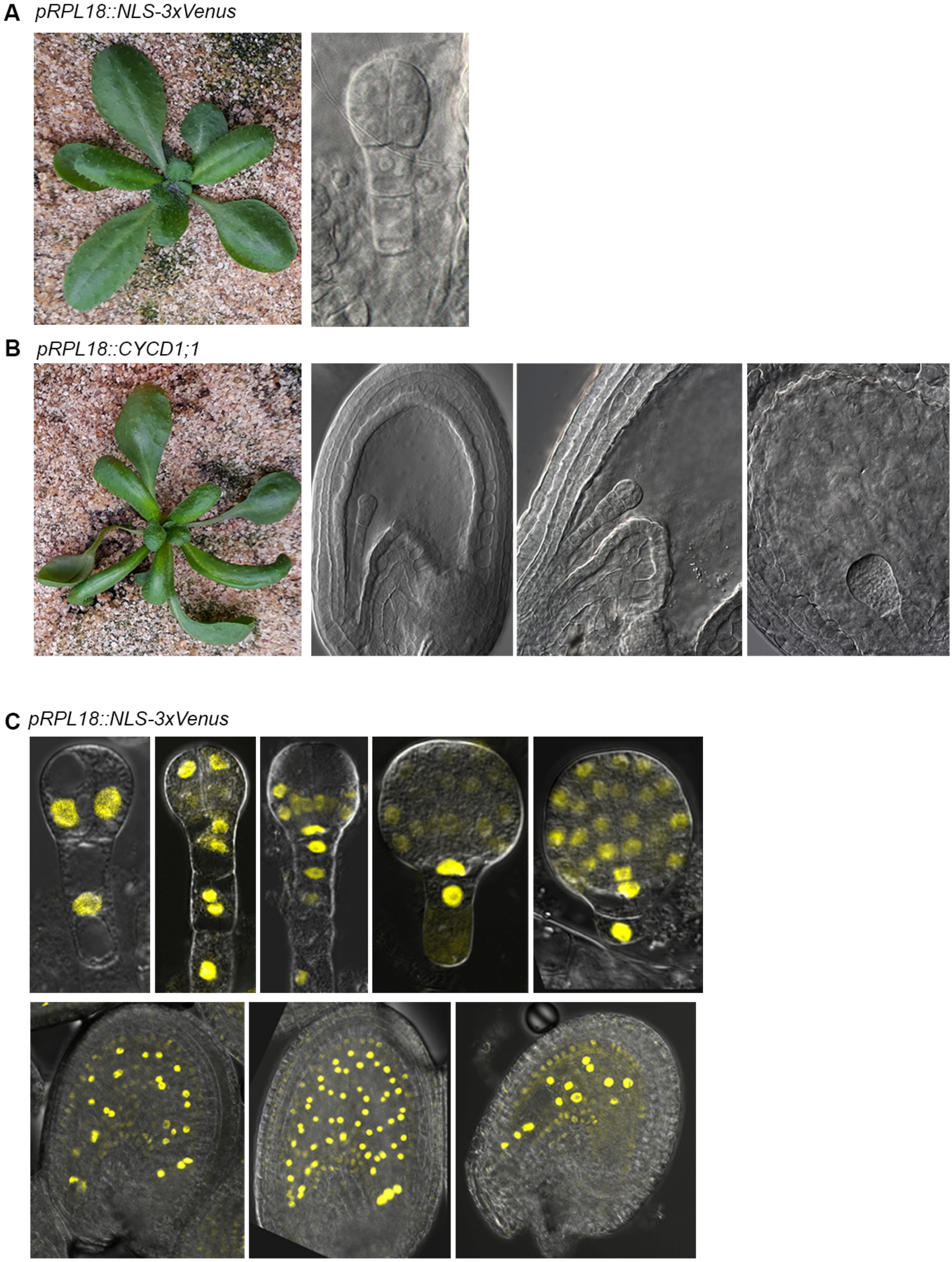

Figure S7. Phenotypes resulting from ectopic expression of *CYCD1;1* under control of the *pRPL18* promoter and reporter gene expression controlled by the latter in embryos and seeds, related to Figure 6.

A. Images of rosettes of *pRPL::NLS-3xVenus* (left) and embryo at globular stage by DIC analysis (right).

B. Images of rosettes of *pRPL18::CYCD1;1* (left), and by DIC microscopic analysis of seeds (right), showing embryos with abnormal shape surrounded by normal endosperm.

C. Confocal imaging of the *pRPL18::3xNLS-Venus* line showing the expression pattern conferred by the *pRPL18* promoter in the developing embryo and seed.

Supplementary Table S2. Primers used in this study related to STAR Methods

	pCYCD1;1::3xNLS-Venus-3'UTR
CYCD1;1 promoter	Fw gtggtctcaGGAGtagtcgcaacttgcaaac<br>rev gtggtctcaCATttctctccccataaccggagatg
CYCD1;1 3'UTR	Fw gtggtctcaGCTTaatttggggagtgaaagtagag<br>Rev gtggtctcaAGCGgatattgaaggagttgttcaatg
	pCYCD1;1::CYCD1;1-GFP-3'UTR
CYCD1;1 promoter	Fw gtggtctcaGGAGtagtcgcaacttgcaaac
	Rev gtggtctcaCATttctctccccataaccggagatg
CYCD1;1 gene	Fw gtggtctctaATGAGGAGTTACCGTTTTAGTG<br>Rev gtggtctcTcaccATTAGAGGTAGATGTTTCATC
CYCD1;1 3'UTR	Fw gtggtctcaGCTTaatttggggagtgaaagtagag<br>Rev gtggtctcaAGCGgatattgaaggagttgttcaatg
	pMEA::MEA-GFP-3'UTR
MEA fragment 1	Fw ggtctcaGGAGtcaggatatgtttaatataacc<br>Rev ggtctcTCACAAAATCcAGACCCTCCA
MEA fragment 2	Fw ggtctcTTGTGAACTGGTGGATGTATTAC<br>Rev ggtctcCAAACGAATGGGGAGGTTAAG
MEA fragment 3	Fw ggtctcCGTTTGGAGGGTCTgAATTTTG
	Rev ggtctcCCATtaaccactcgcctcttc
MEA fragment 4	Fw gtggtctctaATGGAGAAGgttagtttc<br>Rev gtggtctcaaaccGGGCTCATACTTCTCATGCA
GFP fusion with MEA	Fw ggtctcaggttctatggtgagcaagggcgagga<br>Rev ggtctcaggaactcttgtacagctcgtccatgc
MEA fragment 5	Fw gtggtctcattccGAGTCTAGATCCgtaagcatt<br>Rev gtggtctcATGCTCACTGCATGGTTGTC
MEA fragment 6	Fw gtggtctcGAGCATTGTTACCTCAAAgtc<br>Rev GTTTGGATCTGACACCACAA
MEA fragment 7	Fw TATCAAACAAGATTGTGGTGTC<br>Rev gtggtctcTaagCTAACGAGCTGGACGGGCTTC
MEA fragment 8	Fw gtggtctcaGCTTtttgatctgaggagaagcagc<br>Rev gtggtctctAGCGattccacaaatccaac
	pRPL18::CYCD1;1
RPL18 promoter	Fw ggtctcaGGAGattggtgatcaaagtcaatactc<br>Rev ggtctcCCATttttcgtctggagagagac
CYCD1;1 CDS	Fw ggtctctaATGAGGAGTTACCGTTTTAGTG<br>Rev g ggtctcTaagcTTAATTAGAGGTAGATGTTTC
	**CYCD1;1 GABI_214D10 genotyping**
Wild-type band	Fw ATTCATGGCCTGGTGATTCTATC<br>Rev ttacaaagttttcaataaagccga
	**ddPCR**
GFP	Fw GCGGCACGACTTCTTCAAGAG<br>Rev TCGATCCTGTTGACGAGGGT<br>5' FAM- ACGGGAACTACAAGACA –MGB 3' probe
RFP	Fw GACGGCTGCCTCATCTACAAC<br>Rev CCCAGCCGAGTGTTTTCTTC<br>FAM-TGAACTTCCCATCCAAC MGB 3' probe
Internal control (*At5g62150*)	Fw CAACGCTTCTAATTCGATTAGAGGT<br>Rev GGATATGCGGGTTTCGCTC<br>probe VIC ACCATCGGCGATAAA-MGB
CYCD1;1	Fw CGGGTACCTTTCTCGGGTTC<br>Rev TCGCTACACAGAGAATCGCG<br>Rev exonic GAAAGCTCGCTTCTTTTATGTTGG
UBC21	Fw ATGCTTGGAGTCCTGCTTGG<br>Rev TGCCATTGAATTGAACCCTCTC

CUT&RUN and ChIP	
Region -2693	Fw TCAACCAAGACCAAAGTGGA<br>Rev CAAGCACTTGCATGATGAGT
Region -2288	Fw AATGACTCGACCCACTTGTT<br>Rev TAGCATAGTCAACTCGGACC
Region -2149	Fw TCAATAGGGTCCGAGTTGAC<br>Rev TTCATGGGAGCTATTGCCTT
Region -1870 / Region A	Fw AGGTTGGGAGGTGCATATTT<br>Rev TTATGGCTAGGTTGGGCTAC
Region -1226	Fw AAGGCACACGTGAAAGAAAG<br>Rev CCTTTTCGTAGAACCGGAGA
Region -546	Fw CGACCAACAAAATGGTCCAA<br>Rev ACGTTGCATGAGTGAGTACA
Region -336	Fw TCCTTGTTCCTAACCTACGC<br>Rev CGGTTACCGTTTCCGTTATG
Region -75 / Region B	Fw CCTTCACTCTTCTCTCCACA<br>Rev AACCGGAGCTAGATAAACCG
Region 197 / Region C	Fw TGATTTCTCGTCTTCCGAGG<br>Rev ATAGGCTCGCCTTGAGAATC
Region 362	Fw ATTCTCAAGGCGAGCCTATT<br>Rev GCCGTTAAAGGCTGAAAGTT
Region 1570	Fw AGAAGCGAGCTTTCTTGAGT<br>Rev GGGGATTGACAACAGAGGAT
Region 1884	Fw AGCTTCGAGTGAGTGTAAGG<br>Rev CACCTACCCATGAATAGCCA
Region 2057	Fw TGGGGAGTGAAAGTAGAGGA<br>Rev TTCCCCTTGGTTGGAAAACT
Region 2521	Fw TAATGGATCGCAGCAGTTCA<br>Rev GTCACGTGAAGTCACACACA
Region 2925	Fw TTTGATTTTGGTGTGGCCTG<br>Rev TCCACAACACACATGTTCAC
Region 3345 / Region D	Fw CCACGAAACCATAACGTAGC<br>Rev TGGCTAATGTTTGAGGGGTT
Region 3586	Fw CGACCAACAAAATGGTCCAA<br>Rev ACGTTGCATGAGTGAGTACA
Region 4015	Fw ACAAAAGGTTGGCATGGTTT<br>Rev AACTCCATCTGGTTCACGTT
Region 4309	Fw AATGACCAGCCCACACATTT<br>Rev GCTTAGGTCCCTTTGTGTCC
Region 4514	Fw TACACGCGTTTGCCATTAGT<br>Rev TTTGTCTCTACCCCATTCCCA
Mlu-like	Fw GATTTACAAGGAATCTGTTGGTGGT<br>Rev CATAACATAGGTTTAGAGCATCTGC
MEA promoter	Fw ATCGCCCAAGCTTGTGCC<br>Rev GGAGGTTAAGTCTATCCGCCGTAA
PHE1 promoter	Fw TTGTGGTCTTTAGAATCTGATGTTTAG<br>Rev CAGTTGGATAGAAAGTAATGTTATGGC
